# miRglmm: a generalized linear mixed model of isomiR-level counts improves estimation of miRNA-level differential expression and uncovers variable differential expression between isomiRs

**DOI:** 10.1186/s13059-025-03549-y

**Published:** 2025-04-22

**Authors:** Andrea M. Baran, Arun H. Patil, Ernesto Aparicio-Puerta, Seong-Hwan Jun, Marc K. Halushka, Matthew N. McCall

**Affiliations:** 1 Department of Biostatistics and Computational Biology, University of Rochester Medical Center, 265 Crittenden Blvd, Box 630, Rochester, NY 14642 USA; 2 Lieber Institute for Brain Development, Johns Hopkins University, 855 North Wolfe St. Suite 300, Baltimore, MD 21205 USA; 3 Institute of Pathology and Laboratory Medicine, Cleveland Clinic, 9500 Euclid Ave, Cleveland, OH 44195 USA

**Keywords:** microRNA, isomiR, Differential expression, Mixed model, Aggregation, miRNA-seq

## Abstract

MicroRNA-seq data is produced by aligning small RNA sequencing reads of different microRNA transcript isoforms, called isomiRs, to known microRNAs. Aggregation to microRNA-level counts discards information and violates core assumptions of differential expression methods developed for mRNA-seq data. We establish miRglmm, a differential expression method for microRNA-seq data, that uses a generalized linear mixed model of isomiR-level counts, facilitating detection of miRNA with differential expression or differential isomiR usage. We demonstrate that miRglmm outperforms current differential expression methods in estimating differential expression for miRNA, whether or not there is differential isomiR usage, and simultaneously provides estimates of isomiR-level differential expression.

**Supplementary Information:**

The online version contains supplementary material available at 10.1186/s13059-025-03549-y.

## Background

MicroRNA (miRNA) are a class of small, noncoding RNAs that perform a role in transcriptional regulation. They are typically 18–24 nucleotide-long single-stranded RNA molecules that bind to mRNA causing translational suppression or mRNA degradation [1, 2]. Through this mechanism, miRNA can regulate entire pathways and drive disease pathogenesis [3, 4]. The specificity of each miRNA:mRNA interaction leads to discrete downstream consequences [5]. Small RNA sequencing (sRNA-seq) can be used to measure miRNA expression. This process involves enriching samples for small RNA species prior to sequencing, followed by aligning the sequence reads to known miRNAs, tRNA fragments, or other RNA species [6]. The miRNA count data, which we refer to as miRNA-seq data throughout, is produced when sequence reads are aligned to known miRNAs, exclusively.

The specific mature sequence listed in miRNA databases is considered the canonical miRNA sequence [7]. Different isoforms of individual miRNAs, called isomiRs, can arise from both variation in the nucleotide sequence or from variation in the transcript length [8]. Sequence length variants have more or fewer nucleotides at the 5′ and/or the 3′ end of the canonical sequence, whereas polymorphic (internal) isomiRs include different nucleotides within the mature sequence [7]. A third form of isomiR is the addition of an adenosine or uracil tail by a terminal uridylyl transferase (TUT) or similar enzyme [9]. We will use the general term isomiR to describe the various sequence isoforms mapped to a miRNA, without distinction between biologically or technically derived isomiRs, with the assumption that in biological samples most sequences observed at sufficient levels to model are biologically relevant isomiRs.

A collection of isomiRs align to a given miRNA [10]. As an example of isomiR diversity within a miRNA, Additional File 1: Tables S1 and S2 display isomiR-level miRNA-seq read counts for two miRNA: hsa-let-7a-5p and hsa-miR-26a-5p. Typically, read counts of each isomiR for a given miRNA are aggregated by summation and summarized as a single read count for each miRNA (as shown in the last row of Additional File 1: Tables S1 and S2). There are numerous combinations of isomiR counts that produce the same miRNA counts; therefore, this aggregation leads to the loss of any information contained in individual isomiR expression.

IsomiRs are thought to possess unique biological roles [11]. Like canonical sequences, isomiRs are conserved throughout evolution, and biogenesis of isomiRs is tightly regulated. The region of heterogeneity between isomiRs may have unique implications for miRNA-mediated translation regulation [8]. 5’ isomiRs have modifications at the 5’ end of the miRNA resulting from differential processing of paralogous pre-miRNA and can result in regulation of distinct target mRNA [12]. 3’ isomiRs are more common and result from post-transcriptional trimming or tailing sequence modifications [13]. These modifications can alter miRNA function by impacting target recognition or extent of target repression. Additionally, these modifications can impact the stability of the molecule as adenylation protects miRNAs from degradation, while uridylation is associated with increased degradation [7]. Naturally occurring isomiRs have been shown to play distinct roles in a variety of biological processes including cytokine expression, virus proliferation, apoptosis, and tumor progression [11]. Differential expression (DE) at the isomiR-level has identified isomiRs with cancer-specific expression [14]. The biological importance of isomiRs highlights the need for a miRNA-seq analysis method that can account for distinct isomiR expression patterns in estimating miRNA-level differential expression, while also producing more granular estimates of isomiR-level differences.

Similar to differential expression analyses typically performed using bulk mRNA-seq data, the goal of most miRNA-seq studies is to study if, and which, miRNA differ in their expression between groups of samples. Common DE tools developed for mRNA-seq data include DESeq2 [15], edgeR [16], and limma-voom [17]. All of these methods have been frequently used to analyze miRNA-seq data; however, there are some key differences between miRNA-seq data and mRNA-seq data that may make the assumptions of these methods invalid when applied to miRNA-seq data. Due to the compositional nature of RNA sequencing data, the reads can be viewed as a random sample of a fixed size from the pool of all RNA in the library, which can be modeled by a multinomial distribution. In bulk mRNA-seq data, the number of unique mRNA expressed is large and the reads are distributed relatively evenly across the mRNA. In this setting, the features (often genes) can be assumed to be approximately independent. In fact, a negative binomial model for count data, such as that used by DESeq2 and edgeR, may be regarded as a marginalized approximation to an over-dispersed Dirichlet-multinomial model [18]. This approximate independence between features is a key assumption of the tools mentioned above but is violated in miRNA-seq data in two important ways. First, there are generally fewer than 500 miRNA compared to over 10,000 mRNA expressed in a sample, which makes it more likely that random fluctuation in the expression of one miRNA substantially affects the expression of other miRNAs. Second, the distribution of reads in miRNA-seq data is often skewed toward a small number of highly expressed miRNA compared to the more uniform distribution of reads seen in mRNA-seq data [19]. This can induce negative correlation between highly expressed miRNA (due to competition of being counted) regardless of their underlying biological correlation. This also results in data with a small number of highly expressed features making traditional normalization approaches poorly suited for miRNA-seq data. Counts-per-million (CPM) normalization becomes unstable when applied to miRNA-seq data because a small number of miRNAs are responsible for the vast majority of reads in sample. As such, fluctuations in the expression of these miRNAs can have a substantial effect on the total counts and thereby impact the normalized expression of all other miRNAs. Size factor normalization methods generally operate under the assumption of an equal number of features increasing and decreasing in expression across samples (the median ratio method implemented in DESeq2 is an example of this); however, due to the small overall number of miRNAs and the relatively small proportion of overall transcription that they represent, this assumption is unlikely to hold for miRNA-seq data. Differences between miRNA and mRNA make the analysis of miRNA counts at the isomiR level feasible. First, even using Illumina short-read sequencing, miRNAs and all isomiRs are fully sequenced (18–24 nt), whereas typically only fragments of mRNAs (100s of nt out of ~5000–50,000 nt species) are sequenced. Therefore, quantifying the expression of miRNAs based on the entire sequence is possible [6]. Second, as previously stated, there are far fewer miRNA expressed compared to mRNAs. Maintaining and analyzing sequence level data at the scale needed for mRNA analysis would be computationally burdensome. Recent work has highlighted the importance of analyzing miRNA-seq data at the isomiR level [10]. Through the analysis of 28 public miRNA-seq datasets and a newly generated human endothelial cell hypoxia data set, the authors showed substantial differences between isomiR expression and their corresponding canonical miRNAs when applying DESeq2 to miRNA counts versus isomiR counts. As noted above, aggregate miRNA level data violates the assumption of independence between features due to the small number of unique miRNAs expressed in a sample and the highly skewed distribution of miRNA expression. While analyzing isomiR level data greatly increases the feature space and reduces the overall skew of the expression distribution, the isomiR level data introduce a new source of dependence due to high correlation between isomiRs from the same miRNA, which also violates a core assumption of the DESeq2 model. In summary, miRNA are sufficiently different from mRNA, with isomiRs additionally contributing important information, that miRNA-seq-specific analysis pipelines that utilize isomiR-level data are warranted.

Negative binomial mixed models (NBMM) can be used to model overdispersed count data when there is a correlation structure among the counts [20]. This is the case when working with miRNA-seq data at the isomiR level, as counts that are observed for the same isomiR or for the same sample are correlated. Examples of the correlation structure observed between isomiRs of 3 miRNAs are shown in Additional File 1: Fig. S1. Modeling the raw (non-normalized) counts directly has advantages over the typical counts per million (CPM) normalization. CPM is a dependent normalization strategy, so a change in any one miRNA read count will lead to changes in all other miRNA values even in the absence of a change in absolute expression [21]. To account for differences in sequencing depth between samples, one can incorporate an offset term into the NBMM to adjust for the total overall read count within each sample. NBMMs have been proposed for other next-generation sequencing analyses, including RNA-seq, metagenomic sequencing, and single-cell RNA-seq analyses [22–25]. However, the random effects in these models are generally used to model dependence structures arising from longitudinal data where the same organism is measured multiple times or from single-cell data in which many cells come from the same experimental sample. These dependence structures differ from isomiR-level microRNA-seq data in which the dependence arises primarily from the analysis of many isoforms of the same miRNA.

In this manuscript, we propose miRglmm, a method to model isomiR-level counts using a generalized linear mixed model to estimate miRNA-level DE while also obtaining estimates of isomiR DE and differential isomiR usage. We compare miRglmm to several commonly used DE tools on simulated data, an experimental benchmark data set, and real biological data sets. In both simulations and experimental benchmark data, miRglmm has a lower Mean Squared Error (MSE) than other DE tools and better confidence interval coverage. Additionally, we find significant differential isomiR usage exists within most miRNA in real biological data sets, further motivating the use of miRglmm to analyze miRNA-seq data.

## Results

**Fig. 1:**
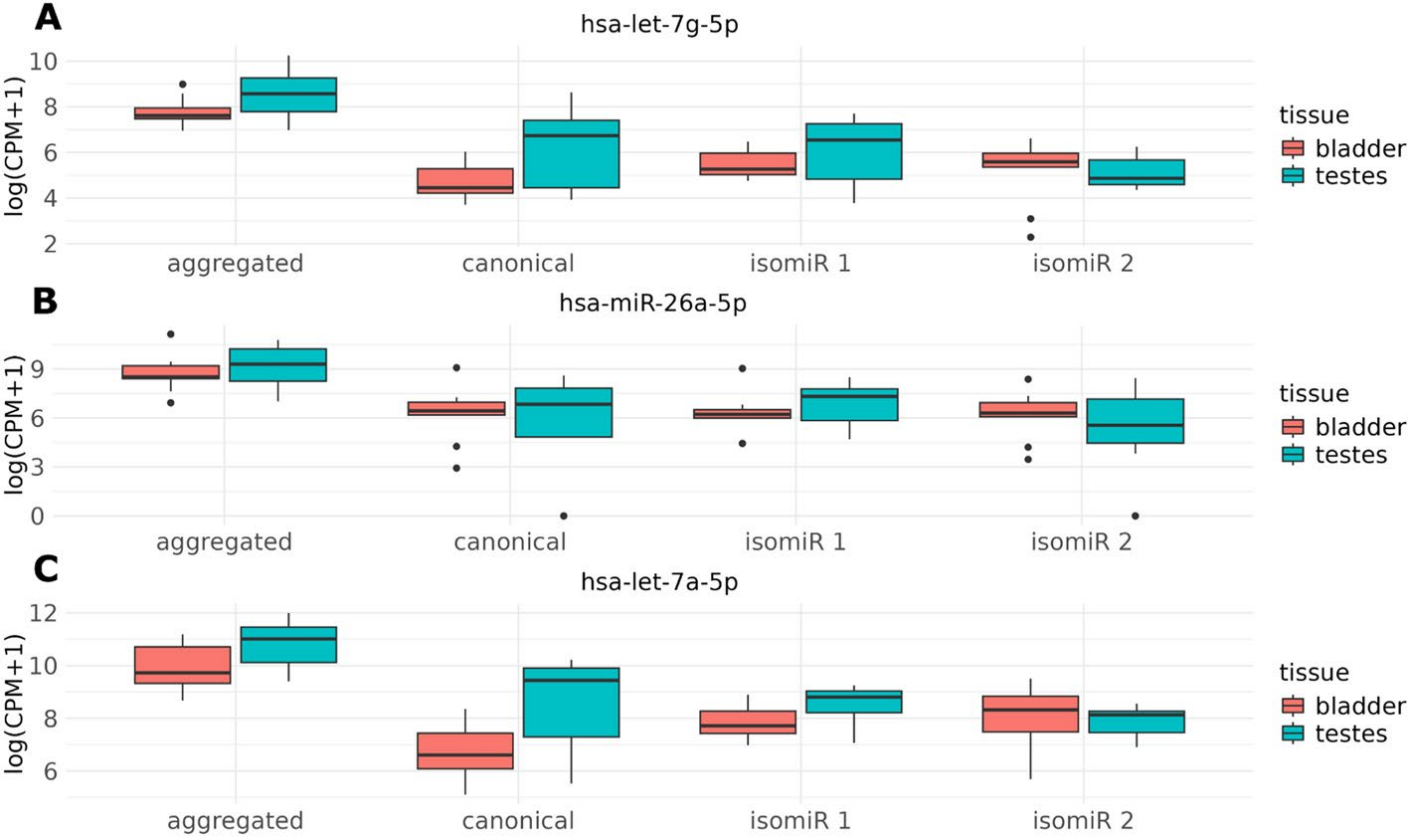
Tissue-specific counts are summarized by boxplots for three miRNAs (panel **A** hsa-let-7g-5p, panel **B** hsa-miR-26a-5p, panel **C** hsa-let-7a-5p). Aggregated miRNA-level counts from summing counts across isomiRs within a miRNA are compared to the canonical/representative sequence and the two highest expressed non-canonical isomiRs (isomiR 1 and isomiR 2). The canonical sequence counts, and isomiR 1 are all higher in testes than bladder, but isomiR 2 has the opposite trend, which is masked when counts are aggregated to miRNA-level. CPM: counts per million

The methodology developed in this manuscript was motivated by two initial observations. First, different isomiRs of the same miRNA can behave very differently between groups of samples. To illustrate this, we selected a study of bladder and testes samples [26], with the goal of minimizing possible technical variation, and therefore capturing true biologically relevant isomiR-level differences. We observed isomiRs with uniformly zero counts within bladder and large non-zero counts in testes, and other isomiRs with uniformly zero counts within testes and large non-zero counts in bladder (Additional File 1: Tables S1 and S2). Aggregation to miRNA counts masks these isomiR differences and results in a loss of information. Additionally, we observed evidence of differential isomiR usage between bladder and testes (Fig. 1). We considered the canonical sequence (which is typically the most highly expressed sequence) and the next two highest expressing isomiRs, as these contribute most to the aggregate miRNA count. Even in this small subset of isomiRs, we observed differences in DE between tissues across isomiRs, indicating the need for a model that can capture this differential isomiR usage.

Second, while miRNA-level data exhibit an artificially induced negative correlation between highly expressed miRNAs, isomiR-level data do not always exhibit this bias. Data generated by high-throughput RNA sequencing is fundamentally compositional in nature. In other words, the resulting reads can be viewed as a random sample of a fixed size from the pool of RNA generated during library preparation. This produces a competition-to-be-counted [27] in which randomly measuring more of one feature decreases the chance of measuring other features. Consider the extreme example of only two features; with a fixed number of total reads, it is clear that a higher count for feature 1 implies a lower count for feature 2, resulting in perfect negative correlation between the two features regardless of their underlying biological correlation. Frequently, this induces a negative correlation between the two most highly expressed miRNAs; however, this induced correlation is not always observed for the most highly expressed isomiRs. To illustrate this, we selected a study of immune cell types [28], with the goal of analyzing samples without cellular heterogeneity that can occur in tissue-level samples. We compared the correlation between the two highest expressed miRNAs with the correlation between the two highest expressed isomiRs for each cell type. While we observed a negative correlation between the highest expressed miRNAs for all cell types, the same was not true at the isomiR level where we observed a mix of positive and negative correlations depending on the cell type (Additional File 1: Fig. S2). This indicates that utilizing the isomiR-level count data could overcome technical biases seen with aggregated miRNA-level count data. To test whether the anti-correlation is primarily a technical artifact, and that highly expressed miRNAs are not generally negatively correlated in the cells themselves, we compared miRNA-seq and qPCR data from the same experiment [29]. There was general positive correlation between the most abundant miRNAs in the two qPCR data sets (Additional File 1: Fig. S3A-B), while the 5 most abundant miRNAs in the miRNA-seq data were frequently negatively correlated with each other (Additional File 1: Fig. S3C).

### miRglmm: a generalized linear mixed effects model for miRNA-seq data analysis

We developed a method and corresponding software to analyze isomiR-level counts from miRNA-seq data. Our method, miRglmm, accounts for dependencies introduced by reads coming from the same sample and from the same isomiR, by using a generalized linear mixed model with random effects for sequence and sample. miRglmm directly models the counts without the need for transformation and includes an offset term to normalize for sequencing depth (see “Methods” for details). miRglmm utilizes a negative binomial mixed model by default, but also provides the option to run a Poisson mixed model (hereafter referred to as miRglmm-Poisson). Unlike existing methods, miRglmm provides estimates of differential expression at both the miRNA level and the isomiR level. miRglmm is implemented in a free and open-source R package available at: https://github.com/mccall-group/miRglmm.

### miRglmm outperforms other methods in detecting differentially expressed miRNAs in the presence of differential isomiR usage

To assess the performance of miRglmm in comparison to existing methods (DESeq2 [15], edgeR [16], limma-voom [17] and a Negative Binomial Generalized Linear Model (NB GLM) [30]), we used a collection of monocyte samples to simulate differential expression at both the miRNA and isomiR level (see “Methods”). Over 100 simulations, miRglmm provided the lowest mean MSE and highest coverage proportion among all the methods (Table 1). Additionally, miRglmm minimized MSE in 96% of simulations (96/100) when comparing methods within a given simulation (Additional File 1: Table S3). DESeq2 and miRglmm-Poisson minimized the MSE in 3 simulations and 1 simulation, respectively. miRglmm provided the most precise logFC estimates for the differentially expressed miRNA, as measured by mean variance of the estimates. While not the smallest, miRglmm also provided similar precision as other methods in null miRNA logFC estimates. All methods (except Wilcoxon testing) exhibited similar power, true positive rate (TPR), and ability to control the type-I error rate, 1-true negative rate (TNR). Additional File 1: Tables S3-S9 provide additional summary statistics for the performance metrics shown in Table 1 across the 100 simulations. Since it operates on isomiR-level data, miRglmm takes longer to run than all other methods, while DESeq2 has the highest memory usage.

**Table 1:** Performance of DE methods across 100 simulations

	Mean MSE (10^−3^)	Mean coverage proportion	Mean null variance (10^−3^)	Mean DE variance (10^−3^)	Mean TPR	Mean TNR	Mean AUC	Time (min)	Memory used (MB)
miRglmm	14.85	0.91	8.45	23.00	0.95	0.96	0.99	5.37	365.2
miRglmm-Poisson	18.75	0.91	11.91	27.23	0.93	0.96	0.98	1.88	273.5
NB GLM	23.15	0.77	8.13	50.64	0.97	0.95	0.99	0.017	105.2
DESeq2	21.92	0.80	8.04	49.72	0.97	0.98	0.99	0.098	1156.8
edgeR	23.15	NA	8.13	50.63	0.96	0.97	0.99	0.004	29.0
Limma-voom	23.10	0.79	8.08	50.45	0.96	0.97	0.99	0.001	8.4
Wilcoxon	NA	NA	NA	NA	0.54	0.97	0.90	0.001	14.5

*MSE* mean squared error, *DE* differentially expressed, *TPR* true positive rate, *TNR* true negative rate, *AUC* area under ROC curve, *NA* not applicable

More detailed summary statistics for these performance metrics are provided in Additional File 1: Tables S3-S9. Time and memory calculated for 1 simulation, with miRglmm using 8 cores

**Fig. 2:**
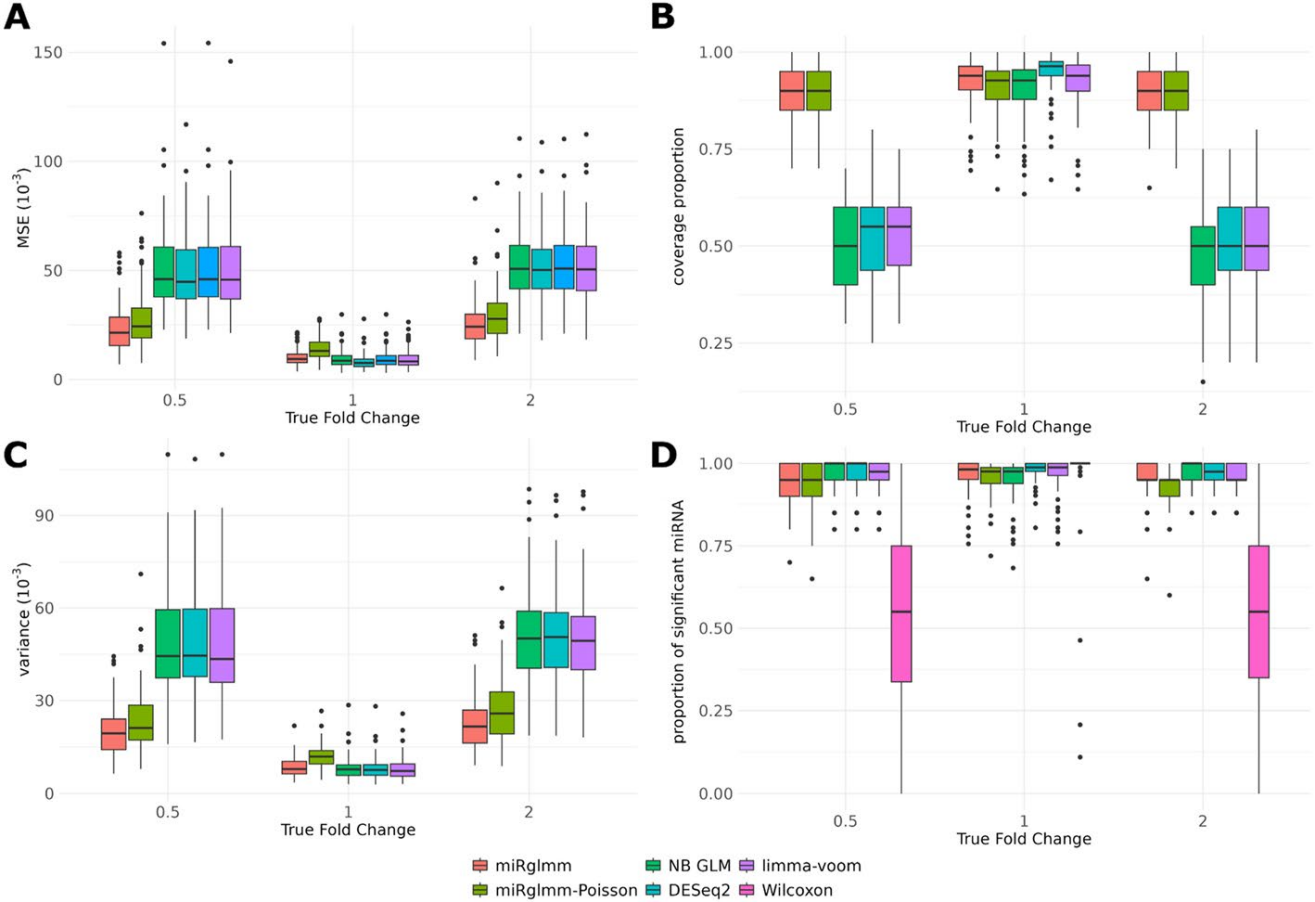
miRglmm and miRglmm-Poisson outperform the aggregation methods in terms of mean squared error (MSE) for miRNA in which an effect is induced (panel **A**). DESeq2 provides the lowest MSE when there is no effect induced. The 95% confidence interval coverage proportion of miRglmm and miRglmm-Poisson are much higher than the coverage proportion of the aggregation methods when an effect is induced (panel **B**). miRglmm and miRglmm-Poisson provide more precise estimates of differential expression compared to aggregation methods (panel **C**). All methods, except Wilcoxon, perform similarly in terms of identifying significant differential expression when it exists (True Fold Change 0.5 or 2) and failing to reject the null when there is no difference (True Fold Change = 1) (panel **D**). edgeR does not provide SE estimates to allow calculation of coverage proportion, and Wilcoxon does not provide effect estimates to calculate MSE, coverage proportion or variance, so these methods are not present in those respective panels. Results are based on 100 independent simulated data sets

We can also assess performance within each “truth” group of miRNA: induced positive effect (*N* = 20), induced negative effect (*N* = 20), or no effect induced (*N* = 82). When we calculated MSE within each group and summarize across simulations, we saw that miRglmm provided markedly lower MSE in the groups with the induced effect compared to all methods operating on the aggregated miRNA-level data, which we will refer to as aggregation methods, and slightly outperformed miRglmm-Poisson (Fig. 2A). In the group of miRNA with no change induced, DESeq2 provided a lower MSE in most simulations. Similarly, the coverage proportion in the two groups of miRNA with induced effects tended to be much closer to the 95% nominal level with miRglmm and miRglmm-Poisson than for the aggregation methods (Fig. 2B). The aggregation methods had very poor confidence interval coverage proportions (0.3–0.4). For the miRNA with no induced effect, DESeq2 tended to have the highest coverage proportion, though miRglmm still provided coverage near the 95% nominal level. miRglmm and miRglmm-Poisson provided more precise estimates of differential expression than the aggregation methods (Fig. 2C). miRglmm-Poisson was less precise in estimating when there is no differential expression, while miRglmm was similar in precision to the aggregation methods. All methods, except Wilcoxon, provided similar ability to identify differential expression when it exists and fail to reject the null hypothesis when there is no differential expression (Fig. 2D).

An additional advantage of miRglmm is that it provides an estimate of the variability in the group effect between isomiRs, facilitating the detection of miRNA with differential isomiR usage between groups. Aggregation methods cannot estimate or account for this variability, and thus do not have the ability to detect miRNAs with differential isomiR usage. We observed that the proportion of miRNAs with significant variability between isomiRs was very high in the groups with the induced effect (Additional File 1: Fig. S4), indicating that our simulation procedure correctly implemented differential isomiR usage. Taken together, these results support the conclusion that aggregation of isomiR counts to miRNA-level counts and the resulting loss of information is detrimental to performance in cases where differential isomiR usage exists. miRglmm, which can account for differential isomiR usage, provides consistently high performance whether or not there is significant differential isomiR usage.

### miRglmm provides accurate estimates of differential expression for isomiRs

Along with providing miRNA-level estimates of differential expression, miRglmm also provides isomiR-level estimates of differential expression. While rarely done, DESeq2 can also provide isomiR-level estimates if run on the isomiR-level count matrix. Summarizing MSE over 100 simulations, miRglmm provided better estimates of isomiR-level differential expression than miRglmm-Poisson or DESeq2 (Additional File 1: Table S10). When we assessed performance within each “true effect” group, we saw that the superior performance of miRglmm holds across all groups. Additionally, isomiR-level estimates of fold change from miRglmm were more precise than estimates from miRglmm-Poisson or DESeq2 (Additional File 1: Table S11). These results contrast with what we observed at the miRNA level, where miRglmm slightly underperformed DESeq2 with respect to MSE and variance when there was no differential expression, but performed much better than DESeq2 when there was either positive or negative differential expression.

### miRglmm maintains high performance under varying simulation scenarios

We assess the effect of varying sample size, effect size, and library size on the performance of miRglmm compared to the other methods using the aforementioned simulation procedure. As sample size decreased from the original *N* = 39, miRglmm minimized MSE across all sample sizes (Additional File 1: Table S12). As expected, as sample size decreased, TPR decreased for all methods, with NB GLM slightly outperforming the other methods (Additional File 1: Table S13). All methods, expect NB GLM, controlled type-I error at or above the nominal level across all sample sizes (Additional File 1: Table S14). Comparison of the area under the ROC curve (AUC) for each method demonstrated clear separation of DE and null miRNA in terms of adjusted *p*-value across all sample sizes for all methods except the Wilcoxon test (Additional File 1: Table S15). Next, we varied the effect size by simulating a fold change = 1.5 and a fold change = 4 to compare to the original simulated effect size of a fold change = 2. miRglmm provided the smallest MSE across all effect sizes, TPR diminished with smaller effect size, TNR indicated type-I errors are controlled, and AUC indicated good separation of null and differentially expressed miRNA (Additional File 1: Tables S16-S19). Varying library size had very little impact on MSE, TPR, TNR, and AUC for all methods (Additional File 1: Tables S20-S23). The performance of miRglmm is also robust to variations in the pre-processing pipeline used (Additional File 1: Tables S24-S26).

### miRglmm outperforms other methods in detecting differentially expressed miRNAs even when there is no differential isomiR usage

While differential isomiR usage is common in real biological data, we wanted to assess how miRglmm would perform in the absence of isomiR variability. To assess the performance of miRglmm in this context, we used a multi-protocol, multi-institution synthetic benchmark dataset originally designed to compare the performance of four different sRNA-seq library preparation methods [31]. This experiment used ratiometric pools of synthesized small RNAs with known variable amounts of differential expression (see “Methods”), providing a known true fold change value for each miRNA that can be used to evaluate the accuracy of DE methods. Because the data came from mixtures of synthesized miRNA pools, there should be no biological variability in isomiR usage.

We used the aforementioned performance metrics to compare DE methods (Table 2). miRglmm accurately estimated the known DE magnitudes between synthetic miRNA pools while maintaining greater than nominal confidence interval coverage, 95% power, and conservative control of the type-I error rate. TPR diminished with effect size for all methods, though miRglmm remained second highest behind NB GLM for all effect sizes (Additional File 1: Table S27). miRglmm provided the most precise estimates as measured by variance across all fold changes (Additional File 1: Table S28). Since miRglmm-Poisson performed far worse than miRglmm, we proceeded with running only standard miRglmm in subsequent analyses of this dataset. In terms of MSE, miRglmm at any isomiR expression filtering threshold provided smaller MSE than all aggregation methods (Additional File 1: Table S29). However, miRglmm with no filtering of lowly expressed isomiRs had the highest MSE, and we observed that the estimates were systemically biased toward the null due to the inclusion of very lowly expressed isomiRs (Additional File 1: Fig. S5A). The four aggregation methods provided similar estimates but underestimated the true effect at all levels. We chose log(median CPM) > -1 as the default filter for miRglmm because this retained the most isomiRs while achieving MSE similar to more stringent filtering thresholds (Additional File 1: Fig. S6). As filtering gets more restrictive, we lose the ability to model some miRNA if fewer than two isomiRs are retained.

**Table 2:** Comparing performance of miRglmm to aggregation methods

	MSE (10^−3^)	Coverage proportion	Null variance (10^−3^)	abs(FC) = 2 variance^a^ (10^−3^)	TPR	TNR	AUC
miRglmm	7.81	0.98	5.01	6.92	0.95	0.97	0.99
miRglmm-Poisson	32.12	0.93	12.95	38.16	0.92	0.94	0.99
NB GLM	12.11	0.97	5.66	9.27	0.96	1.00	1.00
DESeq2	10.19	0.99	5.58	8.00	0.92	1.00	1.00
edgeR	12.11	NA	5.66	9.28	0.90	1.00	1.00
limma-voom	13.78	0.99	6.18	10.66	0.92	1.00	1.00

*MSE* mean squared error, *DE* differentially expressed, *TPR* true positive rate, *TNR* true negative rate, *AUC* area under ROC curve, *NA* not applicable, TPR by abs(FC) is provided in Supplemental Table 27

^a^variance for all FC levels shown in Additional File 1: Table S28

To determine whether other methods would improve if we filtered lowly expressed isomiRs, we compared MSEs based on estimates from running the other methods on aggregated counts from all isomiRs versus filtering prior to aggregation of the isomiR counts. The gain in performance for miRglmm due to filtering was not seen for the aggregation methods (Additional File 1: Fig. S5B). Since aggregating is done via summation, the low counts do not influence the sum to the same degree that they influence the miRglmm estimate.

Due to the synthetic nature of the data, we do not expect any biological isomiRs and presume that the sequence isoforms we are modeling arise solely from technical variation. Accordingly, there was no evidence of differential isomiR usage in the synthetic data (Additional File 1: Fig. S5C). When we removed the extraneous parameter for differential isomiR usage from the model, the performance was similar in terms of the MSE, confidence interval coverage, and bias (Additional File 1: Table S30 and Fig. S7). This indicates that allowing for differential isomiR usage, even if not needed, does not diminish the ability of miRglmm to estimate the effect of interest.

We additionally assessed the performance of miRglmm compared to other methods in the scenario where a significant batch effect exists but is not accounted for in the model. We ran the same analysis as above but did not adjust for Lab. We observed that miRglmm minimized the MSE and had the lowest variance in estimates, but the TPR was significantly lower (Additional File 1: Table S31). The decrease in TPR, coupled with the increase in coverage proportion, suggests that the confidence levels for miRglmm have become wider to account for the unexplained variability in the data. Despite the low TPR, AUC remained high indicating that miRglmm provided good separation between DE and null miRNA, suggesting the selected significance threshold of FDR < 0.05 was leading to the poor performance in terms of TPR.

### Differential miRNA expression and differential isomiR usage in biological samples

We aimed to utilize miRglmm to assess miRNA differential expression, as well as the presence and extent of differential isomiR usage, in real-world biological data. First, we used the dataset from the motivating example shown in Fig. 1 and Additional File 1: Tables S1 and S2. We used miRglmm and other DE methods to compare miRNA expression between bladder (*N* = 9) and testes (*N* = 7). These samples were obtained from one experiment [26], limiting the possibility of technical variation due to library preparation or sequencer differences.

miRglmm detected 15 differentially expressed miRNA between bladder and testes (Additional File 1: Fig. S8 and Table S32). We also have particular interest in identifying miRNA with differential isomiR usage, which is only feasible using miRglmm (Additional File 1: Fig. S8). Four of the 15 differentially expressed miRNA had differential isomiR usage. Importantly, when we considered the miRNA with the largest differential isomiR usage (Additional File 1: Table S33), we found that most of these miRNA were not identified as differentially expressed using miRglmm but were found to be differentially expressed using one or more other DE method. Typically, when miRglmm finds differential isomiR usage, the miRNA log fold change estimate is closer to zero compared to other methods (Additional File 1: Fig. S9). This suggests that aggregation methods are conflating DE of a subset of isomiRs with DE of the miRNA.

**Fig. 3:**
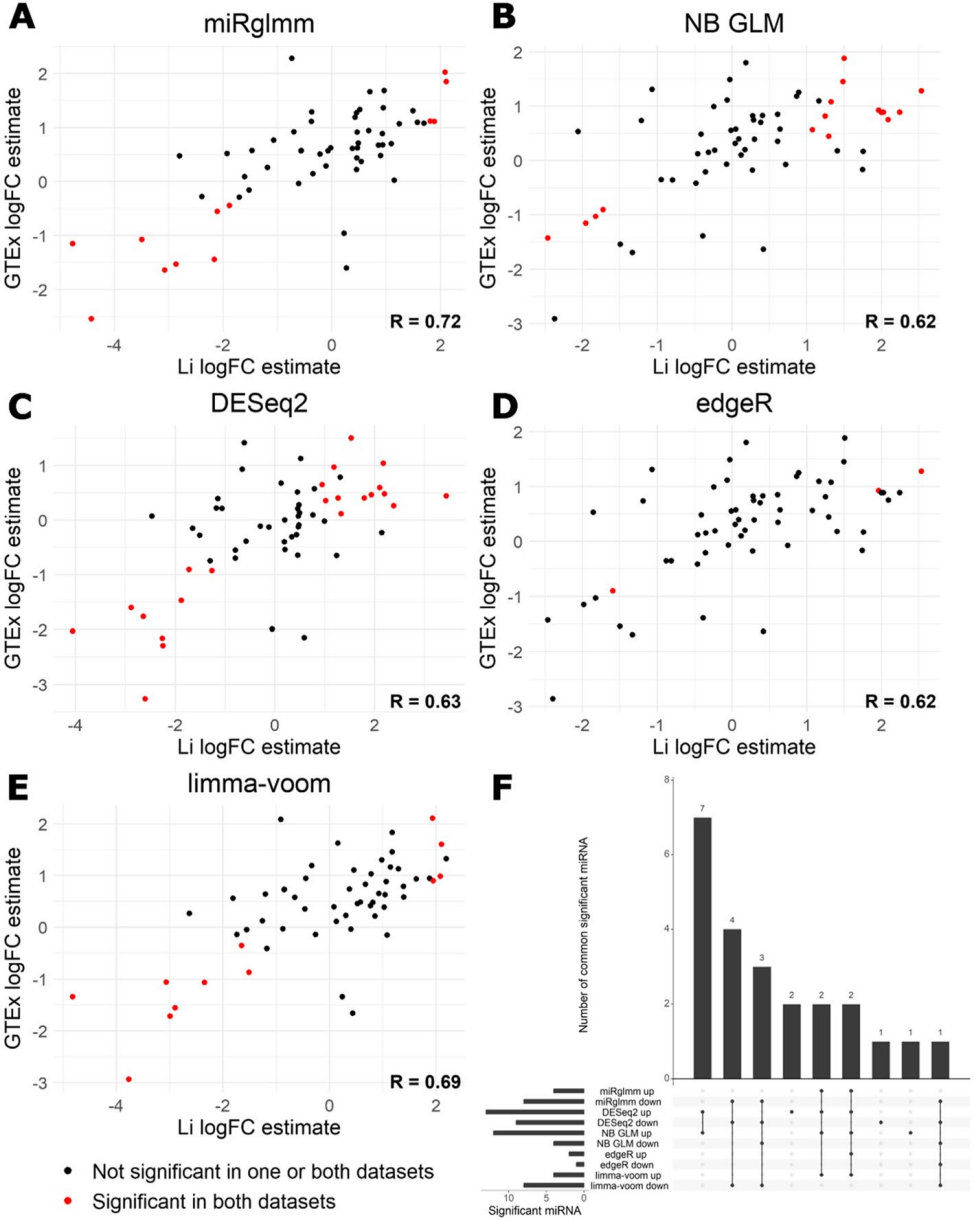
miRglmm (panel **A**) provides a higher Pearson correlation (R) in estimated logFC estimates across datasets than other DE methods (panels **B–E**). miRNA identified as differentially expressed in both datasets are noted by red coloring. The extent of agreement in consistently significant miRNA across methods is shown via an upset plot (panel **F**)

To assess whether miRglmm provides consistent estimates of differential expression, we considered another set of samples of the same tissue types, bladder, and testes. We used bladder (*N* = 32) and testes (*N* = 79) samples from the Genotype-Tissue Expression (GTEx) Project and replicated the above analysis. We found that miRglmm provided a higher correlation in miRNA-level differential expression estimates between datasets than any other method (Fig. 3A–E). We did not quantitatively assess agreement in significant findings between datasets due to the large sample size differences between the datasets, but we did identify miRNA that were identified as differentially expressed in both datasets. We found that 12 of the 15 miRNA identified in our initial miRglmm analysis were consistently identified as differentially expressed in the same direction in the GTEx data (Fig. 3A). When comparing miRNA consistently identified as differentially expressed between datasets across methods, we found that NB GLM and DESeq2 provided the largest set of consistently identified miRNA (Fig. 3F). Of these 7 miRNA, all had significant differential isomiR usage (all FDR < 2 x 10^-5^) that NB GLM and DESeq2 cannot account for. Four of these miRNA (hsa-let-7a, hsa-let-7b, hsa-let-7c, and hsa-miR-146b-5p) were in the list of miRNA with the highest differential isomiR usage (Additional File 1: Table S33, 3 of 4 highlighted in Fig. S9). The use of miRglmm accounts for and estimates differential isomiR usage while other DE methods cannot and provides high correlation in miRNA-level DE estimates between datasets.

We also analyzed a set of immune cell samples from a single study [28]. We used miRglmm to perform differential expression analyses comparing miRNA expression between five immune cell types: monocytes (*N* = 39), Natural Killer Cells (*N* = 38), CD4 + T lymphocytes (*N* = 35), CD8 + T lymphocytes (*N* = 32) and CD19 + B lymphocytes (*N* = 26). We identified many differentially expressed miRNA for each contrast (Additional File 1: Fig. S10), and all miRNA were found to have differential isomiR usage across the 5 cell types. Agreement in the miRNA called differentially expressed between methods was high (Additional File 1: Fig. S11). miRglmm identified several miRNA as differentially expressed that other methods did not (Additional File 1: Table S34). For these miRNA, the estimated differential expression estimates appeared to be greater in magnitude for miRglmm than other methods, as opposed to being similar in size but with a more precise confidence interval. This can occur when a group of lower expressing isomiRs exhibit a consistent difference in expression between cell types that is not seen in the highest (or highly) expressed isomiRs (Additional File 1: Fig. S12). Of the 12 miRNA uniquely found by miRglmm, 11 differed in the same direction between cell types measured via qPCR in a murine immune cell atlas [32] (Additional File 1: Table S34).

## Discussion

With miRglmm, we addressed the need for a DE tool specifically suited for miRNA-seq data that can also utilize information from isomiRs in estimating miRNA-level DE estimates [10]. Commonly used DE tools, such as DESeq2 [15], edgeR [16], and limma-voom [17], were built for analysis of mRNA-seq data, and some key features of miRNA-seq violate the independence between features assumption of these methods. Additionally, these tools run on aggregated miRNA-level counts, resulting in the loss of information contained in individual isomiRs. There is evidence of the biological importance of isomiRs [11], and miRglmm allows for estimation of isomiR-level differential expression that can be used for exploration of individual isomiR effects.

We showed that miRglmm can identify miRNA with differential isomiR usage. When differential isomiR usage exists, miRglmm far outperforms other DE tools in terms of MSE and confidence interval coverage, indicating that miRglmm provides a better estimate of DE at the miRNA level than those produced by other DE tools. Even in settings where differential isomiR usage does not exist, performance of miRglmm remains superior to other DE tools, albeit to a lesser degree. Consistent isomiR expression reduces variability and leads to a precise estimate of miRNA-level differential expression.

In general, we saw a large degree of overlap in miRNA called differentially expressed by miRglmm and other commonly used DE tools (Additional File 1: Fig. S11). MiR-378, miR-150, and miR-223 have been established as monocyte specific via microarray [33] and were also found to be differentially expressed in monocytes vs B cells using miRglmm and all aggregation methods. miRglmm identified 12 miRNA as differentially expressed between immune cell subsets that no other method found (Additional File 1: Table S34). Of these 12 miRNA, 11 show similar DE patterns via qPCR [32], supporting the validity of the unique miRglmm discoveries. Additionally, several of these 12 miRNA (Additional File 1: Table S34) have been previously identified as differential markers. miR-181a is a well-known differential miRNA marker between B lymphocytes and T lymphocytes, exhibiting higher levels in B lymphocytes than T lymphocytes by Northern blot in mice [34], and by Taqman array in humans [35]. miR-191 has been shown to be differentially expressed between B-cell progenitors and T-cell progenitors by microarray and qPCR in mice [36]. By Northern blots, miR-191 is higher in splenic B lymphocytes relative to splenic T lymphocytes [37]. In mice, by Northern blots, miR-26a is modestly higher in splenic B lymphocytes relative to splenic T lymphocytes [37]. By microarray, miR-26a is higher in splenic B lymphocytes [37]. The remainder of miRNA identified as DE only by miRglmm may represent novel discoveries in human immune cells worthy of future investigation.

A limitation of miRglmm is that it does not aim to distinguish a true biological isomiR from a technically arising isomiR and treats all isomiRs of a given miRNA equally. It has been shown that differences in library preparation can induce bias in quantification of isomiRs [38, 39]. By filtering lowly expressed isomiRs, we eliminate some technical variability due to background count alignment. Our study designs in the DE analysis of immune cell types as well as bladder vs testes tissue were implemented to minimize technical variation by selecting samples processed by a single laboratory using consistent library preparation techniques and sequencing equipment. Importantly, we observed the majority of miRNA did have significant differential isomiR usage between cell types / tissues in both data sets where we assume we are capturing primarily biological variability. If we were to analyze larger datasets that encompass multiple studies, we would expect even higher rates of miRNA with significant differential isomiR usage, though some of this would be technical. In this case, miRglmm is flexible and can adjust for technical design variables, such as laboratory, library preparation method, or sequencer, as we did in analyzing the ERCC benchmark data set [31].

## Conclusions

We provide a new method and analysis tool, miRglmm, that uses isomiR variability to improve differential expression analysis of miRNAs from miRNA-seq datasets. miRglmm provides superior performance to alternative DE tools, whether or not significant differential isomiR usage exists, and estimates both miRNA-level and isomiR-level differential expression.

## Methods

### miRglmm: a generalized linear mixed model (GLMM)-based DE tool for miRNA-seq data

The statistical model implemented in miRglmm is as follows. Let ${C}_{ij}$ denote the count for sample $i=1,...,n$ and isomiR $j=1,...,J$. Let ${T}_{i}=\sum_{j=1}^{J}{C}_{ij}$ be the total counts for sample $i$. This is used as an offset term in the model to adjust for variable sequencing depth across samples. For each sample $i$, let ${X}_{i}$ be a $p$-vector of covariates. For each miRNA, miRglmm fits the following negative binomial mixed model (NBMM):

$${C}_{ij}\sim \text{NB}\left({\mu }_{ij},\Theta \right)$$

$$log\left({\mu }_{ij}\right)=log\left({T}_{i}\right)+\left(\beta +{\tau }_{1j}\right){X}_{i}+{\tau }_{i}+{\tau }_{0j}$$

$$\left[\begin{array}{c}{\tau }_{0j}\\ {\tau }_{1j}\end{array}\right]\sim N\left(0,{\Omega }_{i}\right),{\Omega }_{i}=\left[\begin{array}{cc}{\sigma }_{0j}^{2}& {\sigma }_{01j}\\ {\sigma }_{01j}& {\sigma }_{1j}^{2}\end{array}\right]$$

$${\tau }_{i}\sim N\left(0,{\sigma }_{1}^{2}\right)$$

$\beta$ is the fixed effect of primary interest. ${\tau }_{i}$ is the random intercept term for sample $i$, ${\tau }_{0j}$ is the random intercept for isomiR $j$, and ${\tau }_{1j}$ is the random slope term for isomiR $j$. The random effects for sample and isomiR are independent, while the intercept and slope random effects for isomiR have a dependence structure specified by $\Omega$. The variance of the random slope ${\sigma }_{1j}^{2}$ can be used to assess variable DE between isomiRs. We built miRglmm using the function glmer.nb in the lme4 package, which fits an NBMM via a Laplace approximation to the maximum likelihood with a variety of optimizer choices [40]. All mentions of log() refer to natural logarithms, which is the default of the log function in R. miRglmm also provides the option of fitting a Poisson mixed model, instead of the NBMM fit by default.

### Software, data structures, outputs, and reproducibility

miRglmm is an R library that consists of one core function that can be easily integrated into DE analysis pipelines by replacing DE methods designed for mRNA-seq (such as DESeq2, edgeR, limma-voom). miRglmm is implemented in a free and open-source R package available at: https://github.com/mccall-group/miRglmm. IsomiR-level count matrices in the form of core Bioconductor structures, SummarizedExperiment objects, are taken as input, and a list of model fit summaries for each miRNA analyzed is returned. The function can be run in parallel across miRNA and can handle flexible design matrices. The miRglmm package vignette includes examples of how to import isomiR-level count data from either miRge [41] or sRNAbench [42]. The analyses presented in this paper are reproducible using code in the GitHub repository found at https://github.com/mccall-group/miRglmm_paper. This repository includes functions to extract and summarize values of interest from the model fit summaries, including miRNA-level estimates of DE with confidence intervals, isomiR-level estimates of expression within miRNA, and estimates of differential isomiR usage within miRNA with the associated likelihood ratio test.

### The microRNAome data resource

The miRNAome dataset was assembled to more fully understand miRNA expression patterns across primary cell types [43, 44]. The dataset was built upon 2077 samples from 175 public datasets across 196 primary cell types. miRNA annotation and quantification was performed using the miRge3.0 pipeline [41]. Briefly, miRge3.0 is a multi-step miRNA alignment program. From a FASTQ file, miRge3.0 collapses identical sequences and processes them through repeated Bowtie alignment steps to identify canonical miRNAs, isomiRs, and other RNA species. In addition to a final read count per miRNA, it outputs an alignment file containing counts of all aligned isomiRs, which was used here.

### Filtering of lowly expressed miRNAs and isomiRs

Counts per million (CPM) normalization of aggregated counts were used to assess overall miRNA expression, with the goal of retaining miRNA with sufficient expression to model. A threshold of log(median CPM) > 5 was used to retain miRNA for modeling. Even after filtering at the miRNA level, the resulting count matrix contains very sparse isomiR-level counts so miRglmm also filters lowly expressed isomiRs that contribute low/no amount of information to the model. Specifically, CPM normalization of isomiR-level counts was used to assess isomiR expression, and an isomiR filter based on log(median CPM) is implemented as an input argument of miRglmm (default = -1).

### Inducing known effects in real biological data to simulate differential expression

We created ground truth data by inducing a known artificial effect into real biological data, allowing us to assess the performance of miRglmm under conditions seen in real data. We searched the miRNAome data [43] for one cell type with large sample counts coming from the same study to have a relatively homogenous starting dataset. We used 39 monocyte samples from one study [28] and retained 122 miRNA with sufficient expression to model using a log(median CPM) cutoff of 5, as described above.

To induce an artificial “group” effect, we randomly split the samples into 2 groups, with 19 samples labelled as Group A and 20 samples labelled as Group B (Additional File 1: Fig. S13). Of 122 total miRNA, we used stratified sampling to select 20 miRNA to be overexpressed in Group A, and another 20 miRNA to be underexpressed in Group A. The sampling was stratified by total miRNA expression to manipulate miRNA across the full range of expression values. We used the default log(median CPM) cutoff of -1 to retain isomiRs for analysis. For the 20 miRNA overexpressed in Group A, we multiplied the counts of all retained isomiRs by a random truncated normal variable with mean value of 2, variance of 1, lower bound of 1, and upper bound of 3 in Group A samples only. These 20 miRNAs now have a known miRNA-level fold change (B vs A) = 0.5, with differential isomiR usage. For the 20 miRNA underexpressed in Group A, we multiplied the counts of all retained isomiRs by a random truncated normal variable with the same parameters as above but this time in Group B samples only. These 20 miRNAs now have a known miRNA-level fold change (B vs A) = 2, with differential isomiR usage. Importantly, we recalculated the total counts used in the offset term after the artificial signal is induced. The stratification used in sampling miRNA ensures the effect of the signal being added is consistent across groups, even though the total counts increase for all samples. The entire procedure, including randomly splitting samples into two groups, was repeated 100 times.

This simulation procedure was also used to assess the effect of varying sample size, effect size, and library size on performance. To vary sample size, we took subsets of the 39 samples to achieve desired sample sizes per group. To vary effect size, we multiply counts by a random truncated normal variable with mean value of 1.5, variance of 1, lower bound of 1, and upper bound of 2 for the fold change = 1.5 analysis, and multiply the counts by a random truncated normal variable with mean value of 4, variance of 1, lower bound of 3, and upper bound of 5 for the fold change = 4 analysis. To vary library size, we subsample 50 and 75% of the original counts (100% library size).

For each miRNA, we estimated a miRNA-level differential group effect, measured via log fold-change (logFC) estimates, using miRglmm and miRglmm-Poisson. We also produced differential group effects using commonly used differential expression tools: DESeq2 [15], edgeR [16], and limma-voom [17]. We included a Negative Binomial Generalized Linear Models (NB GLM), fit using glm.nb from the MASS R package [30], which is similar to the miRglmm model but without random effects that can model differential isomiR usage. We also performed differential expression testing using the Wilcoxon rank-sum test, though the Wilcoxon method does not produce estimates of group effects. Data was aggregated to the miRNA-level prior to running NB GLM, DESeq2, edgeR, limma-voom, and Wilcoxon, and hence we collectively call these methods “aggregation methods.” Aggregated count values were produced by summing counts from all isomiRs of a given miRNA.

We used mean squared error (MSE) and 95% confidence interval coverage proportions to assess accuracy. The MSE compared the estimated logFC to the induced effect for each miRNA, and the coverage proportion assessed the proportion that the true logFC fell in the 95% confidence interval estimated by the model. We cannot estimate a confidence interval coverage proportion for edgeR as that algorithm does not provide standard error (SE) estimates. We used the variance of the estimates to assess precision. We calculated the variance of all differentially expressed miRNA logFC estimates (which we refer to as DE variance), and separately, calculated the variance of all null (no effect added) miRNA logFC estimates (which we refer to as null variance). We used true positive rate (TPR) to assess the ability to detect differential expression where it exists (i.e., statistical power). We used true negative rate (TNR) to assess the ability to control type-1 error. To calculate TPR and TNR, we first adjusted the *p*-values of the group effect using Bejamini Hochberg False Discovery Rate and considered miRNA with FDR < 0.05 differentially expressed. We calculated TPR as the proportion of significant miRNA among those where we induced an effect for TPR. We calculated TNR as the proportion of non-significant miRNA among those where we did not induce an effect. We also used area under the ROC curve, to assess the ability to distinguish differentially expressed miRNA from miRNA with no group difference, regardless of any one FDR threshold. Where appropriate, we also separately looked at each method’s performance metrics when the effect was induced up or down, and where no effect was induced.

To identify miRNA with differential isomiR usage, we tested whether the random slope parameter τ_1j_ in the miRglmm model contributes significant information with a 1-degree of freedom (1-df) likelihood ratio test (LRT) comparing likelihoods from the model specified above and a model removing only the τ_1j_ parameter. With this test, we can identify miRNA with differential isomiR usage (i.e., significant random slope effects) and summarize by induced effect groups.

### Analysis of a benchmark experiment using synthetic miRNA pools

We used experimental benchmark data with known expression differences to assess the performance of miRglmm, miRglmm-Poisson, and existing methods to estimate DE. The Extracellular RNA Communication Consortium (ERCC) was established to facilitate expansion of the field of extracellular RNA (exRNA) biology and consisted of collaborative projects to develop robust methods for isolation and analysis of exRNA data [45]. One of these projects was a multi-protocol, multi-institution assessment of the bias of four sRNA-seq library preparation methods, using ratiometric pools of synthesized small RNAs [31]. Chemically synthesized RNA oligonucleotides were added in varying ratios to pool A and pool B, from 10 to 1 and 1 to 10, leading to 15 levels of DE (from logFC = -2.3 to logFC = 2.3), providing a known fold change value for each miRNA that can be used to evaluate the accuracy of miRglmm and other methods (Additional File 1: Fig. S14).

The ERCC sequence runs with 4N method (4 random nucleotides on both ends of the reads) were chosen (*n* = 104 runs). The reference sequence database consisting of 286 human miRNAs and 48 other spikein miRNAs were indexed using bowtie-index. The reads were processed for Illumina adapters 'TGGAATTCTCGGGTGCCAAGGA' using cutadapt [46] followed by alignment using bowtie aligner [47]. The parameters for bowtie include "No mismatch (-n 0)", "Trim 4 nucleotides on both ends (-5 4 -3 4)", "Avoid alignment against the reverse-complement reference strand (-norc)," and output in SAM format (-S). The SAM files were processed for read counts across each mapped miRNA account for PCR duplicates (4N-based) using custom Python scripts. The miRNA counts were used for the downstream analyses.

The ERCC data was processed by 6 different laboratories (Lab), and we used dimensionality reduction using non-metric multidimensional scaling (NMDS) to assess if there was sufficient laboratory variability to require adjustment for Lab in the analysis. The samples separated along the first dimension by Lab, and the second dimension appeared to capture the Pool effect (Additional File 1: Fig. S15A). We included a fixed effect for Lab in the models for all methods to adjust for the technical effect of the differences in lab-specific sample handling, processing, and sequencer on the counts. Due to the need for adjusting for Lab, we did not run the Wilcoxon method for this analysis. The ERCC dataset included synthetic RNAs that were much longer at 50–90 nucleotides than the typical 18–24 nucleotide miRNAs. We excluded any RNA specified to have a length > 45, resulting in a set of 303 small RNAs.

This synthetic dataset also provided justification for establishing filters at both the miRNA and isomiR level. Since the oligonucleotides were synthetically added, we expect all included miRNA to be expressed. The distribution of miRNA expression via log(median CPM) of aggregated counts in this data can be used to determine a suitable threshold for miRNA expression, supporting our choice of log(median CPM) > 5 used in all analyses. To filter sequences, we aimed to separate biologically relevant isomiR counts from random background expression. Since the ERCC contains sequence isoforms that do not map to known miRNA, we can consider these background counts and compare the distribution of expression levels to isomiR counts that map to known miRNA to identify an appropriate range for an isomiR-level filter. When we compared the distribution of background sequence expression (reads not mapping to any known miRNA) to expression of sequences that map to miRNA, we saw that the distributions separated around a log(median CPM) value of -1 (Additional File 1: Fig. S15B). We ran miRglmm without any sequence filtering, and also compared the performance of miRglmm under a range of reasonable filters (log(median CPM) > -1 (default) to 2). We also assessed the effect of filtering prior to aggregation when using the aggregation methods.

For each miRNA, we estimated a differential Pool effect, adjusted for Lab and measured via logFC estimates, using miRglmm under a variety of isomiR filters. We also aggregated the data to the miRNA count level and ran the aforementioned aggregation methods for comparison. We used MSE and 95% confidence interval coverage proportions to assess the performance as described above. We utilized a LRT as previously described to assess if there was significant differential isomiR usage.

### Differential expression analyses of real biological data of tissues and cell types

Our goal in selecting samples for these analyses was to minimize possible technical variation, and therefore capture true biologically relevant isomiR-level differences. We chose bladder and testes tissues for this analysis because they represented a large set of tissues available from a single study [26], where sample processing and sequencer would be consistent across samples. We used bladder and testes samples from the Genotype-Tissue Expression (GTEx) Project as a comparator dataset. Additionally, we chose a set of immune cell types that were also derived from a single study as our cell type differential expression analysis set [28]. We aimed to produce miRNA-level differential expression (DE) estimates using miRglmm and compare these results to other commonly used differential expression tools that run on aggregated miRNA-level count data.

For each miRNA, we estimated a miRNA-level differential group (tissue or cell type) effect, measured via log fold-change (logFC) estimates, and assessed significance, using miRglmm. We utilized a LRT as previously described to identify miRNA that have significant differential isomiR usage. We can produce isomiR-level estimates of expression within group by summing fixed and random effects for each isomiR.

## Supplementary Information

Additional File 1: Supplemental figures and tables

Additional File 2: miRglmm software package vignette

## Data Availability

Biological sample metadata used in this manuscript were accessed through the microRNAome database (43, 44). The original raw count data for the specific studies analyzed can be accessed via the sequence read archive (SRA) under the following accession numbers: SRP110505 (48) and SRP007946 (49). Synthetic data used in this manuscript were accessed via SRA accession number SRP098949 (31). Source code for miRglmm is available on GitHub (50) under the MIT license, and on Zenodo (51). Processed data files used in this manuscript can be found on Zenodo (52). Codes to reproduce all analyses and figures in this manuscript are also available on GitHub (53), under the MIT license, and on Zenodo (54). The GTEx data used for the analyses described in this manuscript were obtained from: the GTEx Portal on 11/25/2024 and dbGaP accession number phs000424.v10.p2 on 11/25/2024 (55).
