# Supplementary material for: miRglmm: a generalized linear mixed model of isomiR-level counts improves estimation of miRNA-level differential expression and uncovers variable differential expression between isomiRs
Source: Genome Biol. 2025 Apr 22;26:102. doi: 10.1186/s13059-025-03549-y (PMC12016310; doi:10.1186/s13059-025-03549-y)
Supplement: Supplementary file 1 — Additional File 1: Supplemental figures and tables [file 13059_2025_3549_MOESM1_ESM.docx]

Table S1: Example of isomiR data for miRNA hsa-let-7a-5p

|  | SRR333658 | SRR333672 | SRR333674 | SRR333678 | SRR333680 | SRR333682 |
| --- | --- | --- | --- | --- | --- | --- |
|  | bladder | bladder | bladder | testes | testes | testes |
| **TGAGGTAGTAGGTTGTATAGTT*** | **768** | **321** | **461** | **1988** | **3463** | **1534** |
| TGAGGTAGTAGGTTGT | 2216 | 2269 | 1869 | 322 | 186 | 244 |
| TGAGGTAGTAGGTTGTATAG | 1327 | 812 | 1060 | 587 | 1169 | 605 |
| TGAGGTAGTAGGTTGTATAGTTT | 86 | 35 | 43 | 2173 | 2312 | 890 |
| TGAGGTAGTAGGTTGTATAGT | 1169 | 481 | 764 | 602 | 1000 | 310 |
| TGAGGTAGTAGGTTGTATA | 990 | 722 | 762 | 175 | 334 | 157 |
| TGAGGTAGTAGGTTGTATAGTTTCG | 0 | 0 | 0 | 789 | 973 | 492 |
| TGAGGTAGTAGGTTGTATAGTTTC | 25 | 11 | 9 | 535 | 616 | 839 |
| TGAGGTAGTAG**A**TTGTATAGTTTCG | 0 | 0 | 1 | 51 | 1451 | 493 |
| TGAGGTAGTAGGTTGTATAGT**A**TCTC | 447 | 431 | 947 | 0 | 0 | 0 |
| TGAGGTAGTAGGTTGTATAGTTAT | 400 | 303 | 336 | 229 | 283 | 145 |
| TGAGGTAGTAGGTTGTATAGT**A**TCT | 414 | 370 | 688 | 0 | 0 | 1 |
| TGAGGTAGTAGGTTGTATAGTTATCT | 668 | 258 | 477 | 0 | 0 | 5 |
| TGAGGTAGTAGGTTGTATAGT**A**TC | 248 | 239 | 521 | 32 | 54 | 50 |
| TGAGGTAGTAGGTTGTATAGT**A**T | 223 | 229 | 453 | 59 | 75 | 32 |
| TGAGGTAGTAGGTTGTATAGTTCGT | 1 | 0 | 0 | 140 | 420 | 174 |
| TGAGGTAGTAGGTTGTATAGTTTT | 1 | 0 | 1 | 192 | 225 | 92 |
| TGAGGTAGTAGGTTGTATAGT**C**GTAT | 3 | 0 | 0 | 29 | 175 | 14 |
| TGAGGTAGTAGG**C**TGT | 17 | 87 | 69 | 0 | 0 | 0 |
| TGAGGTAGTAGGTTGTATAGT**C**GT | 0 | 1 | 0 | 23 | 97 | 15 |
| TGAGGTAGTAGGTTGTATAG**A**TCTC | 35 | 42 | 56 | 0 | 0 | 0 |
| TGAGGTAGTAGGTTGT**G**TAGT | 46 | 21 | 11 | 32 | 16 | 6 |
| TGAGGTAG**G**AGGTTGTATAGT**A**TCT | 37 | 29 | 38 | 0 | 0 | 0 |
| TGAGGTAGTAGGTTGTATAG**A**TCT | 20 | 38 | 42 | 0 | 0 | 0 |
| **hsa-let-7a-5p (aggregated)** | **13141** | **9185** | **12254** | **9626** | **15328** | **9461** |

*denotes canonical/representative miRNA sequence, red text denote internal modifications leading to isomiRs

Table S2: Example of isomiR data for miRNA hsa-miR-26a-5p

| Sequence Read Archive Number | SRR333662 | SRR333664 | SRR333674 | SRR333680 | SRR333684 | SRR333688 |
| --- | --- | --- | --- | --- | --- | --- |
| Tissue Type | bladder | bladder | bladder | testes | testes | testes |
| **TTCAAGTAATCCAGGATAGGCT*** | **303** | **660** | **388** | **163** | **1171** | **383** |
| TTCAAGTAATCCAGGATAGGCTA | 330 | 544 | 412 | 7 | 650 | 231 |
| TTCAAGTAATCCAGGATAGGCTATCG | 0 | 0 | 0 | 4 | 257 | 102 |
| TTCAAGTAATCCAGGATAGGCTATCT | 297 | 227 | 300 | 0 | 0 | 0 |
| TTCAAGTAATCCAGGATAGGCTATCTC | 179 | 226 | 305 | 0 | 0 | 0 |
| TTCAAGTAATCCAGGATAGGCTC | 7 | 7 | 7 | 1360 | 356 | 101 |
| TTCAAGTAATCCAGGATAGGCTCG | 0 | 0 | 2 | 39 | 177 | 48 |
| TTCAAGTAATCCAGGATAGGCTCGT | 0 | 0 | 0 | 190 | 63 | 15 |
| TTCAAGTAATCCAGGATAGGCTCGTA | 1 | 0 | 0 | 8 | 98 | 22 |
| TTCAAGTAATCCAGGATAGGCTT | 0 | 7 | 5 | 139 | 93 | 53 |
| TTCAAGTAATCCAGGATAGGCTTCG | 0 | 0 | 0 | 80 | 45 | 33 |
| TTCAAGTAATCCAGGATAGGCTTCGT | 0 | 0 | 0 | 17 | 35 | 21 |
| TTCAAGTAATCCAGGAT | 76 | 287 | 115 | 16 | 417 | 123 |
| TTCAAGTAATCCAGGATA | 163 | 497 | 164 | 80 | 673 | 233 |
| TTCAAGTAATCCAGGATAG | 161 | 559 | 154 | 115 | 976 | 358 |
| TTCAAGTAATCCAGGATAGG | 236 | 823 | 248 | 189 | 762 | 346 |
| TTCAAGTAATCCAGGATAGGAT | 5 | 20 | 10 | 1 | 1 | 0 |
| TTCAAGTAATCCAGGATAGGATCT | 12 | 23 | 17 | 0 | 0 | 0 |
| TTCAAGTAATCCAGGATAGGATCTC | 14 | 21 | 15 | 0 | 0 | 0 |
| TTCAAGTAATCCAGGATAGGC | 273 | 854 | 421 | 69 | 722 | 328 |
| TTCAAGTAATCCAGGATAGGCA | 10 | 17 | 7 | 6 | 17 | 24 |
| TTCAAGTAATCCAGGATAGGCAG | 1 | 4 | 2 | 1 | 15 | 10 |
| TTCAAGTAATCCAGGATAGGCATCT | 15 | 13 | 6 | 0 | 0 | 0 |
| TTCAAGTAATCCAGTATAGGC | 12 | 1 | 1 | 0 | 1 | 2 |
| **hsa-miR-26a-5p (aggregated)** | **2827** | **6118** | **3452** | **2814** | **8277** | **3386** |

*denotes canonical/representative miRNA sequence, red text denote internal modifications leading to isomiRs


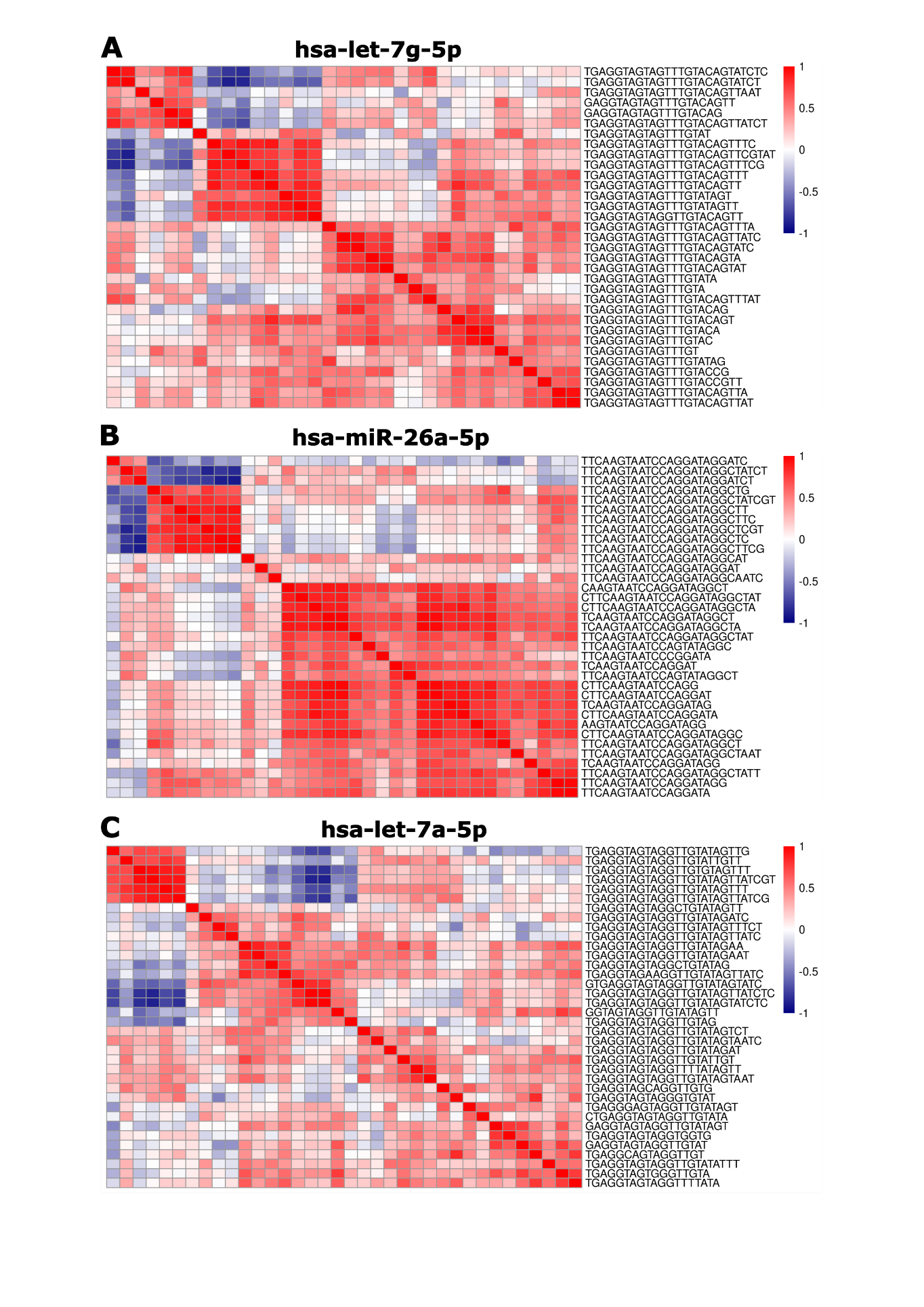


Fig. S1: Pairwise Pearson correlations between isomiRs for 3 miRNAs: (A) let-7g-5p, (B) miR-26a-5p, and (C) let-7a-5p were calculated across the bladder and testes samples from Chen et al. (Gene Expression Omnibus accession GSE31616).


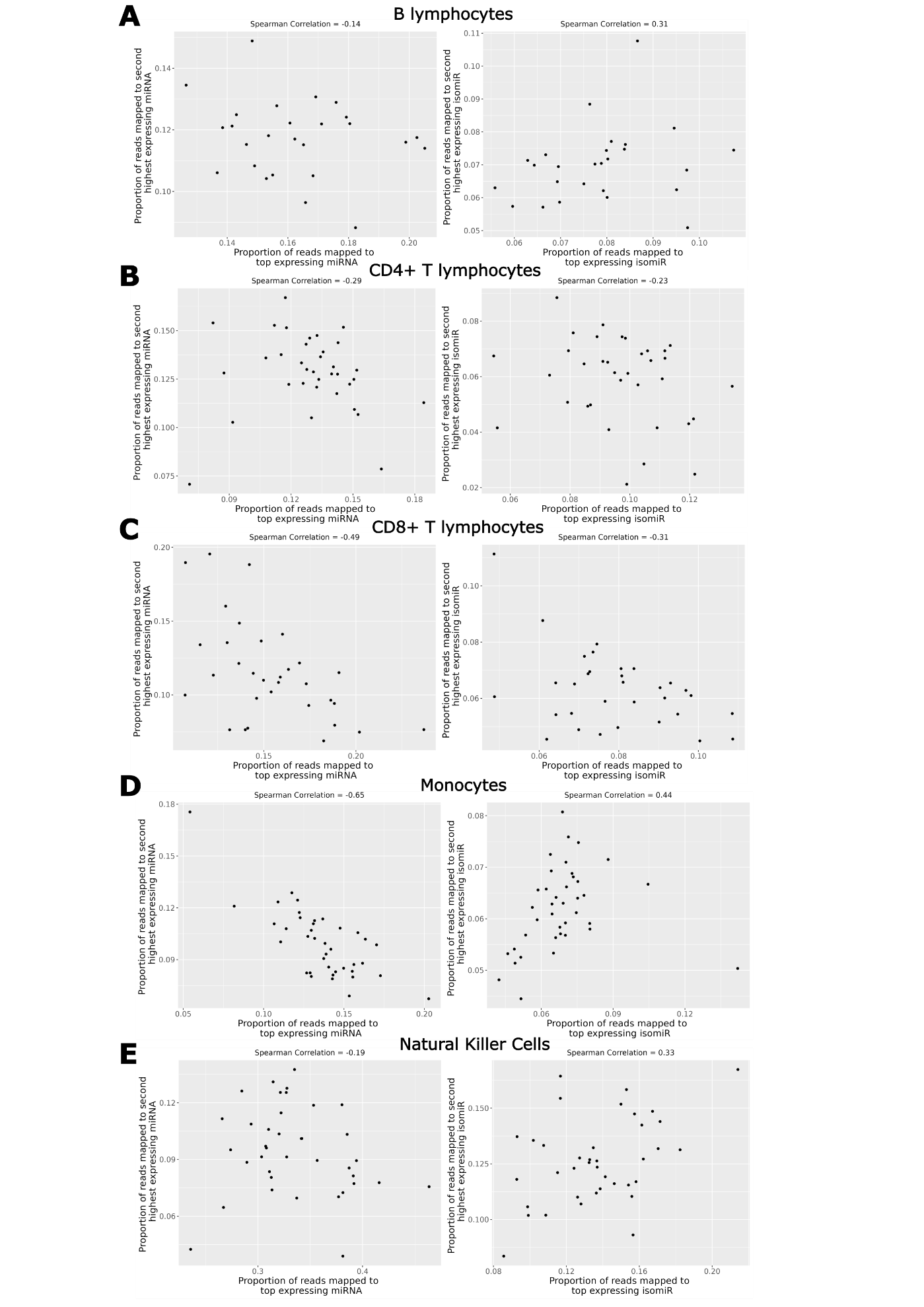


Fig. S2: Negative correlation is observed when considering the top two highest expressing miRNA after aggregation to miRNA-level counts (left column, all panels), whereas a mix of positive (right column, panels A, D and E) and negative correlations (right column, panels B and C) are observed when considering the two highest expressing isomiRs.


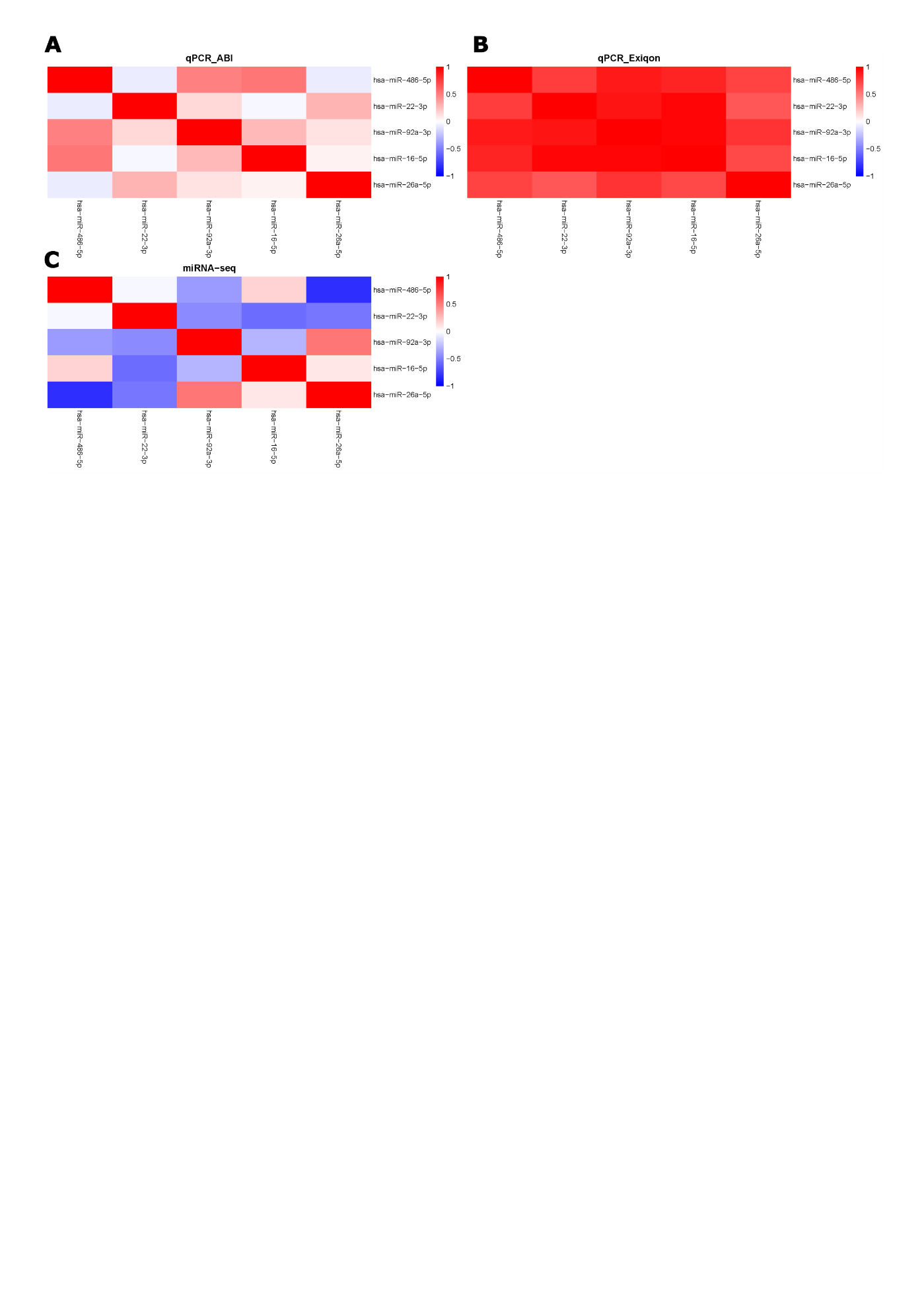


Fig. S3: Negative correlation bias seen in miRNA-seq data (panel C) is not observed when assessing correlation via qPCR (panels A and B), indicating the negative correlations seen arise due to technical biases in sequencing and not due to actual negative correlation in expression.

Table S3: MSE(10^-3^) summary statistics across 100 simulations

| method | mean | SD | median | minimum | maximum | number of  times minimizing MSE |
| --- | --- | --- | --- | --- | --- | --- |
| miRglmm | 14.85 | 4.65 | 13.63 | 7.62 | 32.92 | 96 |
| miRglmm-Poisson | 18.75 | 5.38 | 17.60 | 9.09 | 38.81 | 1 |
| NB GLM | 23.15 | 6.12 | 22.39 | 13.26 | 48.83 | 0 |
| DESeq2 | 21.92 | 5.11 | 21.53 | 12.29 | 36.08 | 3 |
| edgeR | 23.15 | 6.12 | 22.39 | 13.25 | 48.83 | 0 |
| Limma-voom | 23.10 | 6.22 | 22.34 | 13.81 | 45.37 | 0 |

Table S4: Confidence interval coverage proportion summary statistics across 100 simulations

| method | mean | SD | median | minimum | maximum |
| --- | --- | --- | --- | --- | --- |
| miRglmm | 0.91 | 0.06 | 0.93 | 0.71 | 0.98 |
| miRglmm-Poisson | 0.91 | 0.05 | 0.92 | 0.67 | 0.99 |
| NB GLM | 0.77 | 0.06 | 0.79 | 0.53 | 0.89 |
| DESeq2 | 0.80 | 0.04 | 0.81 | 0.66 | 0.89 |
| Limma-voom | 0.79 | 0.07 | 0.80 | 0.55 | 0.89 |

Table S5: Summary statistics for variance (10^-3^) of null miRNA logFC estimates across 100 simulations

| method | mean | SD | median | minimum | maximum |
| --- | --- | --- | --- | --- | --- |
| miRglmm | 8.45 | 2.94 | 7.92 | 3.54 | 21.83 |
| miRglmm-Poisson | 11.91 | 3.54 | 11.93 | 4.45 | 26.63 |
| NB GLM | 8.13 | 3.49 | 7.80 | 3.03 | 28.52 |
| DESeq2 | 8.04 | 3.48 | 7.62 | 2.91 | 28.14 |
| edgeR | 8.13 | 3.50 | 7.81 | 3.04 | 28.54 |
| Limma-voom | 8.08 | 3.60 | 7.29 | 3.07 | 25.75 |

Table S6: Summary statistics for variance (10^-3^) of differentially expressed miRNA logFC estimates across 100 simulations

| method | mean | SD | median | minimum | maximum |
| --- | --- | --- | --- | --- | --- |
| miRglmm | 23.00 | 8.06 | 21.84 | 11.58 | 54.82 |
| miRglmm-Poisson | 27.23 | 9.65 | 25.48 | 12.23 | 67.39 |
| NB GLM | 50.64 | 13.30 | 49.29 | 26.01 | 103.86 |
| DESeq2 | 49.72 | 12.11 | 49.22 | 27.30 | 84.83 |
| edgeR | 50.63 | 13.29 | 49.13 | 25.91 | 103.92 |
| Limma-voom | 50.45 | 13.25 | 49.13 | 26.11 | 99.20 |

Table S7: True Positive Rate summary statistics across 100 simulations

| method | mean | SD | median | minimum | maximum |
| --- | --- | --- | --- | --- | --- |
| miRglmm | 0.95 | 0.04 | 0.95 | 0.80 | 1.00 |
| miRglmm-Poisson | 0.93 | 0.04 | 0.93 | 0.73 | 1.00 |
| NB GLM | 0.97 | 0.03 | 0.98 | 0.85 | 1.00 |
| DESeq2 | 0.97 | 0.03 | 0.98 | 0.90 | 1.00 |
| edgeR | 0.96 | 0.03 | 0.98 | 0.85 | 1.00 |
| Limma-voom | 0.96 | 0.03 | 0.98 | 0.88 | 1.00 |
| Wilcoxon | 0.54 | 0.09 | 0.55 | 0.30 | 0.83 |

Table S8: True Negative Rate summary statistics across 100 simulations

| method | mean | SD | median | minimum | maximum |
| --- | --- | --- | --- | --- | --- |
| miRglmm | 0.96 | 0.05 | 0.98 | 0.76 | 1.00 |
| miRglmm-Poisson | 0.96 | 0.05 | 0.98 | 0.72 | 1.00 |
| NB GLM | 0.95 | 0.06 | 0.98 | 0.68 | 1.00 |
| DESeq2 | 0.98 | 0.03 | 0.99 | 0.80 | 1.00 |
| edgeR | 0.97 | 0.05 | 0.99 | 0.79 | 1.00 |
| Limma-voom | 0.97 | 0.05 | 0.99 | 0.76 | 1.00 |
| Wilcoxon | 0.97 | 0.13 | 1.00 | 0.11 | 1.00 |

Table S9: Area under ROC Curve (ROC) summary statistics across 100 simulations

| method | mean | SD | median | minimum | maximum |
| --- | --- | --- | --- | --- | --- |
| miRglmm | 0.99 | 0.01 | 1.00 | 0.91 | 1.00 |
| miRglmm-Poisson | 0.98 | 0.02 | 0.99 | 0.87 | 1.00 |
| NB GLM | 0.99 | 0.01 | 0.99 | 0.92 | 1.00 |
| DESeq2 | 0.99 | 0.01 | 1.00 | 0.94 | 1.00 |
| edgeR | 0.99 | 0.01 | 0.99 | 0.92 | 1.00 |
| Limma-voom | 0.99 | 0.01 | 1.00 | 0.92 | 1.00 |
| Wilcoxon | 0.90 | 0.12 | 0.96 | 0.53 | 1.00 |


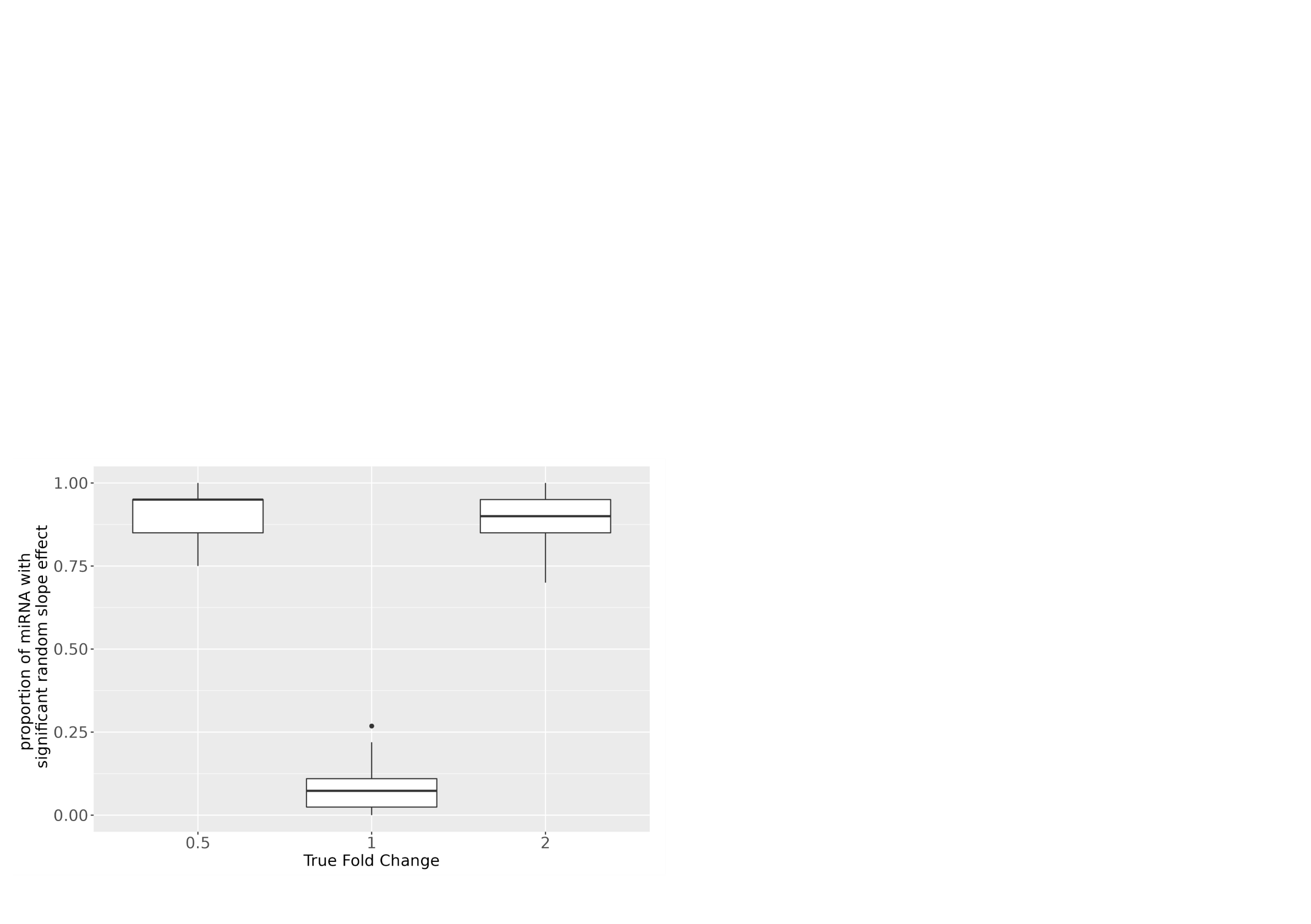


Fig. S4: The simulation procedure creates significant differential isomiR usage.

Table S10: Mean MSE (10^-3^), overall and by true FC, at isomiR-level across 100 simulations

| Method | Overall | FC=0.5 | FC=1 | FC=2 |
| --- | --- | --- | --- | --- |
| miRglmm | 21.59 | 34.99 | 15.11 | 35.21 |
| miRglmm-Poisson | 43.48 | 60.87 | 36.30 | 55.67 |
| DESeq2 | 41.12 | 44.21 | 39.60 | 44.32 |

Table S11: Mean variance (10^-3^) of isomiR-level estimates across 100 simulations

| Method | Null miRNA | DE miRNA |
| --- | --- | --- |
| miRglmm | 12.87 | 88.33 |
| miRglmm-Poisson | 33.58 | 123.97 |
| DESeq2 | 39.16 | 128.51 |

DE: differentially expressed

Table S12: Effect of varying sample size on mean MSE (10^-3^) across 100 simulations

|  | N=10 | N=20 | N=30 | N=39 |
| --- | --- | --- | --- | --- |
| miRglmm | 42.41 | 23.68 | 17.36 | 14.85 |
| miRglmm-Poisson | 50.05 | 29.48 | 21.89 | 18.75 |
| NB GLM | 48.08 | 30.66 | 25.42 | 23.15 |
| DESeq2 | 46.00 | 29.38 | 24.37 | 21.92 |
| edgeR | 48.08 | 30.67 | 25.43 | 23.15 |
| Limma-voom | 47.76 | 30.59 | 25.28 | 23.10 |

Table S13: Effect of varying sample size on mean True Positive Rate across 100 simulations

|  | N=10 | N=20 | N=30 | N=39 |
| --- | --- | --- | --- | --- |
| miRglmm | 0.75 | 0.88 | 0.93 | 0.95 |
| miRglmm-Poisson | 0.70 | 0.84 | 0.91 | 0.93 |
| NB GLM | 0.85 | 0.92 | 0.95 | 0.97 |
| DESeq2 | 0.82 | 0.92 | 0.96 | 0.97 |
| edgeR | 0.77 | 0.91 | 0.95 | 0.96 |
| Limma-voom | 0.77 | 0.90 | 0.95 | 0.96 |
| Wilcoxon | 0.01 | 0.15 | 0.37 | 0.54 |

Table S14: Effect of varying sample size on mean True Negative Rate across 100 simulations

|  | N=10 | N=20 | N=30 | N=39 |
| --- | --- | --- | --- | --- |
| miRglmm | 0.95 | 0.97 | 0.97 | 0.96 |
| miRglmm-Poisson | 0.96 | 0.96 | 0.96 | 0.96 |
| NB GLM | 0.93 | 0.95 | 0.96 | 0.95 |
| DESeq2 | 0.98 | 0.98 | 0.98 | 0.98 |
| edgeR | 0.99 | 0.98 | 0.98 | 0.97 |
| Limma-voom | 0.98 | 0.98 | 0.98 | 0.97 |
| Wilcoxon | 0.99 | 0.98 | 0.99 | 0.97 |

Table S15: Effect of varying sample size on mean Area Under ROC Curve (AUC) across 100 simulations

|  | N=10 | N=20 | N=30 | N=39 |
| --- | --- | --- | --- | --- |
| miRglmm | 0.94 | 0.98 | 0.99 | 0.99 |
| miRglmm-Poisson | 0.93 | 0.97 | 0.98 | 0.98 |
| NB GLM | 0.95 | 0.98 | 0.99 | 0.99 |
| DESeq2 | 0.96 | 0.98 | 0.99 | 0.99 |
| edgeR | 0.96 | 0.98 | 0.99 | 0.99 |
| Limma-voom | 0.96 | 0.98 | 0.99 | 0.99 |
| Wilcoxon | 0.60 | 0.81 | 0.87 | 0.90 |

Table S16: Effect of varying effect size on mean MSE (10^-3^) across 100 simulations

|  | FC=1.5 | FC=2 | FC=4 |
| --- | --- | --- | --- |
| miRglmm | 13.01 | 14.85 | 13.12 |
| miRglmm-Poisson | 17.05 | 18.75 | 17.06 |
| NB GLM | 16.37 | 23.15 | 14.42 |
| DESeq2 | 15.58 | 21.92 | 11.99 |
| edgeR | 16.38 | 23.15 | 14.43 |
| Limma-voom | 16.26 | 23.10 | 14.47 |

Table S17: Effect of varying effect size on mean True Positive Rate across 100 simulations

|  | FC=1.5 | FC=2 | FC=4 |
| --- | --- | --- | --- |
| miRglmm | 0.76 | 0.95 | 1.00 |
| miRglmm-Poisson | 0.71 | 0.93 | 1.00 |
| NB GLM | 0.85 | 0.97 | 1.00 |
| DESeq2 | 0.85 | 0.97 | 1.00 |
| edgeR | 0.83 | 0.96 | 1.00 |
| Limma-voom | 0.83 | 0.96 | 1.00 |
| Wilcoxon | 0.13 | 0.54 | 1.00 |

Table S18: Effect of varying effect size on mean True Negative Rate across 100 simulations

|  | FC=1.5 | FC=2 | FC=4 |
| --- | --- | --- | --- |
| miRglmm | 0.98 | 0.96 | 0.94 |
| miRglmm | 0.97 | 0.96 | 0.94 |
| miRglmm-Poisson | 0.96 | 0.95 | 0.92 |
| NB GLM | 0.98 | 0.98 | 0.98 |
| DESeq2 | 0.98 | 0.97 | 0.95 |
| edgeR | 0.98 | 0.97 | 0.95 |
| Limma-voom | 0.97 | 0.97 | 0.98 |

Table S19: Effect of varying effect size on mean Area Under ROC Curve (AUC) across 100 simulations

|  | FC=1.5 | FC=2 | FC=4 |
| --- | --- | --- | --- |
| miRglmm | 0.96 | 0.99 | 1.00 |
| miRglmm-Poisson | 0.94 | 0.98 | 1.00 |
| NB GLM | 0.96 | 0.99 | 1.00 |
| DESeq2 | 0.97 | 0.99 | 1.00 |
| edgeR | 0.96 | 0.99 | 1.00 |
| Limma-voom | 0.96 | 0.99 | 1.00 |
| Wilcoxon | 0.71 | 0.90 | 1.00 |

Table S20: Effect of varying library size on mean MSE (10^-3^) across 100 simulations

|  | 50% | 75% | 100% |
| --- | --- | --- | --- |
| miRglmm | 14.90 | 15.15 | 14.85 |
| miRglmm-Poisson | 18.17 | 18.81 | 18.75 |
| NB GLM | 23.11 | 23.13 | 23.15 |
| DESeq2 | 21.89 | 21.88 | 21.92 |
| edgeR | 23.12 | 23.13 | 23.15 |
| Limma-voom | 23.09 | 23.08 | 23.10 |

Table S21: Effect of varying library size on mean True Positive Rate across 100 simulations

|  | 50% | 75% | 100% |
| --- | --- | --- | --- |
| miRglmm | 0.95 | 0.95 | 0.95 |
| miRglmm-Poisson | 0.94 | 0.93 | 0.93 |
| NB GLM | 0.97 | 0.97 | 0.97 |
| DESeq2 | 0.97 | 0.97 | 0.97 |
| edgeR | 0.96 | 0.96 | 0.96 |
| Limma-voom | 0.96 | 0.96 | 0.96 |
| Wilcoxon | 0.55 | 0.54 | 0.54 |

Table S22: Effect of varying library size on mean True Negative Rate across 100 simulations

|  | 50% | 75% | 100% |
| --- | --- | --- | --- |
| miRglmm | 0.96 | 0.96 | 0.96 |
| miRglmm-Poisson | 0.96 | 0.96 | 0.96 |
| NB GLM | 0.95 | 0.95 | 0.95 |
| DESeq2 | 0.98 | 0.98 | 0.98 |
| edgeR | 0.97 | 0.97 | 0.97 |
| Limma-voom | 0.97 | 0.97 | 0.97 |
| Wilcoxon | 0.98 | 0.98 | 0.97 |

Table S23: Effect of varying library size on mean Area Under ROC Curve (AUC) across 100 simulations

|  | 50% | 75% | 100% |
| --- | --- | --- | --- |
| miRglmm | 0.99 | 0.99 | 0.99 |
| miRglmm-Poisson | 0.99 | 0.98 | 0.98 |
| NB GLM | 0.99 | 0.99 | 0.99 |
| DESeq2 | 0.99 | 0.99 | 0.99 |
| edgeR | 0.99 | 0.99 | 0.99 |
| Limma-voom | 0.99 | 0.99 | 0.99 |
| Wilcoxon | 0.90 | 0.90 | 0.90 |

Table S24: Performance summary across 100 simulations using miRge without MIREC option in pre-processing

|  | mean MSE (10^-3^) | mean coverage proportion | mean null variance (10^-3^) | mean DE variance (10^-3^) | mean TPR | mean TNR | mean AUC |
| --- | --- | --- | --- | --- | --- | --- | --- |
| miRglmm | 15.66 | 0.90 | 8.91 | 23.09 | 0.95 | 0.95 | 0.99 |
| miRglmm-Poisson | 20.01 | 0.90 | 12.91 | 27.24 | 0.92 | 0.95 | 0.98 |
| NB GLM | 24.34 | 0.76 | 8.40 | 52.16 | 0.96 | 0.94 | 0.99 |
| DESeq2 | 22.52 | 0.80 | 8.37 | 50.74 | 0.97 | 0.98 | 0.99 |
| edgeR | 24.34 | NA | 8.41 | 52.15 | 0.96 | 0.96 | 0.99 |
| Limma-voom | 23.95 | 0.78 | 8.38 | 51.48 | 0.96 | 0.97 | 0.99 |
| Wilcoxon | NA | NA | NA | NA | 0.53 | 0.99 | 0.91 |

MSE: Mean Squared Error, DE: differentially expressed, TPR: True Positive Rate, TNR: True Negative Rate, AUC: Area under ROC Curve, NA: not applicable.

Table S25: Performance summary across 100 simulations using sRNAbench with default parameters in pre-processing

|  | mean MSE (10^-3^) | mean coverage proportion | mean null variance (10^-3^) | mean DE variance (10^-3^) | mean TPR | mean TNR | mean AUC |
| --- | --- | --- | --- | --- | --- | --- | --- |
| miRglmm | 11.10 | 0.92 | 8.22 | 14.42 | 0.98 | 0.97 | 1.00 |
| miRglmm-Poisson | 13.03 | 0.92 | 10.12 | 16.25 | 0.97 | 0.97 | 0.99 |
| NB GLM | 18.37 | 0.81 | 8.28 | 40.57 | 0.98 | 0.96 | 0.99 |
| DESeq2 | 17.67 | 0.83 | 8.26 | 40.21 | 0.98 | 0.98 | 0.99 |
| edgeR | 18.37 | NA | 8.28 | 40.56 | 0.97 | 0.98 | 0.99 |
| Limma-voom | 18.35 | 0.82 | 8.26 | 40.49 | 0.97 | 0.98 | 0.99 |
| Wilcoxon | NA | NA | NA | NA | 0.54 | 0.98 | 0.90 |

MSE: Mean Squared Error, DE: differentially expressed, TPR: True Positive Rate, TNR: True Negative Rate, AUC: Area under ROC Curve, NA: not applicable.

Table S26: Performance summary across 100 simulations using sRNAbench with parameter alignType=v in pre-processing

|  | mean MSE (10^-3^) | mean coverage proportion | mean null variance (10^-3^) | mean DE variance (10^-3^) | mean TPR | mean TNR | mean AUC |
| --- | --- | --- | --- | --- | --- | --- | --- |
| miRglmm | 15.39 | 0.91 | 9.48 | 23.72 | 0.95 | 0.97 | 0.99 |
| miRglmm-Poisson | 21.67 | 0.91 | 15.43 | 30.12 | 0.92 | 0.96 | 0.98 |
| NB GLM | 22.68 | 0.79 | 8.34 | 51.75 | 0.96 | 0.96 | 0.99 |
| DESeq2 | 21.69 | 0.81 | 8.29 | 51.73 | 0.96 | 0.98 | 0.99 |
| edgeR | 22.68 | NA | 8.34 | 51.75 | 0.96 | 0.98 | 0.99 |
| Limma-voom | 22.65 | 0.80 | 8.38 | 51.57 | 0.95 | 0.98 | 0.99 |
| Wilcoxon | NA | NA | NA | NA | 0.54 | 0.98 | 0.92 |

MSE: Mean Squared Error, DE: differentially expressed, TPR: True Positive Rate, TNR: True Negative Rate, AUC: Area under ROC Curve, NA: not applicable.

Table S27: True Positive Rate (TPR) as function of abs(FC)

|  | 1.5 | 2 | 3 | 4 | 5 | 8 | 10 |
| --- | --- | --- | --- | --- | --- | --- | --- |
| miRglmm | 0.73 | 0.92 | 1.00 | 1.00 | 1.00 | 1.00 | 1.00 |
| miRglmm-Poisson | 0.58 | 0.90 | 1.00 | 1.00 | 1.00 | 1.00 | 1.00 |
| NB GLM | 0.75 | 0.95 | 1.00 | 1.00 | 1.00 | 1.00 | 1.00 |
| DESeq2 | 0.55 | 0.92 | 1.00 | 1.00 | 1.00 | 1.00 | 1.00 |
| edgeR | 0.48 | 0.87 | 1.00 | 1.00 | 1.00 | 1.00 | 1.00 |
| limma-voom | 0.63 | 0.87 | 0.97 | 1.00 | 1.00 | 1.00 | 1.00 |

Table S28: Variance (10^-3^) of estimated logFC as a function of abs(FC)

|  | 1.5 | 2 | 3 | 4 | 5 | 8 | 10 |
| --- | --- | --- | --- | --- | --- | --- | --- |
| miRglmm | 6.77 | 6.92 | 2.72 | 9.24 | 9.23 | 12.62 | 8.07 |
| miRglmm-Poisson | 24.04 | 38.16 | 26.67 | 45.09 | 37.10 | 34.56 | 18.95 |
| NB GLM | 13.76 | 9.27 | 7.47 | 19.59 | 13.51 | 13.16 | 9.10 |
| DESeq2 | 12.16 | 8.00 | 5.69 | 17.04 | 10.10 | 12.45 | 8.88 |
| edgeR | 13.77 | 9.28 | 7.47 | 19.59 | 13.51 | 13.16 | 9.10 |
| limma-voom | 12.29 | 10.66 | 8.74 | 20.73 | 18.55 | 15.86 | 10.75 |

Table S29: Comparing performance by miRglmm filters

| method | MSE (x 10^-3^) | number of  miRNA estimated | minimum number  of isomiRs | maximum number  of isomiRs | coverage  proportion |
| --- | --- | --- | --- | --- | --- |
| miRglmm filter -1* | 7.81 | 303 | 7 | 40 | 0.98 |
| miRglmm filter -0.5 | 7.80 | 303 | 6 | 33 | 0.97 |
| miRglmm filter 0 | 8.45 | 303 | 4 | 30 | 0.97 |
| miRglmm filter 0.5 | 8.71 | 302 | 2 | 24 | 0.98 |
| miRglmm filter 1 | 8.58 | 301 | 2 | 22 | 0.98 |
| miRglmm filter 1.5 | 9.05 | 301 | 2 | 18 | 0.99 |
| miRglmm filter 2 | 10.31 | 300 | 2 | 15 | 0.99 |
| miRglmm no filter | 15.34 | 303 | 12 | 263 | 0.97 |

MSE: Mean Squared Error, NA: not applicable, *denotes default filter implemented in miRglmm


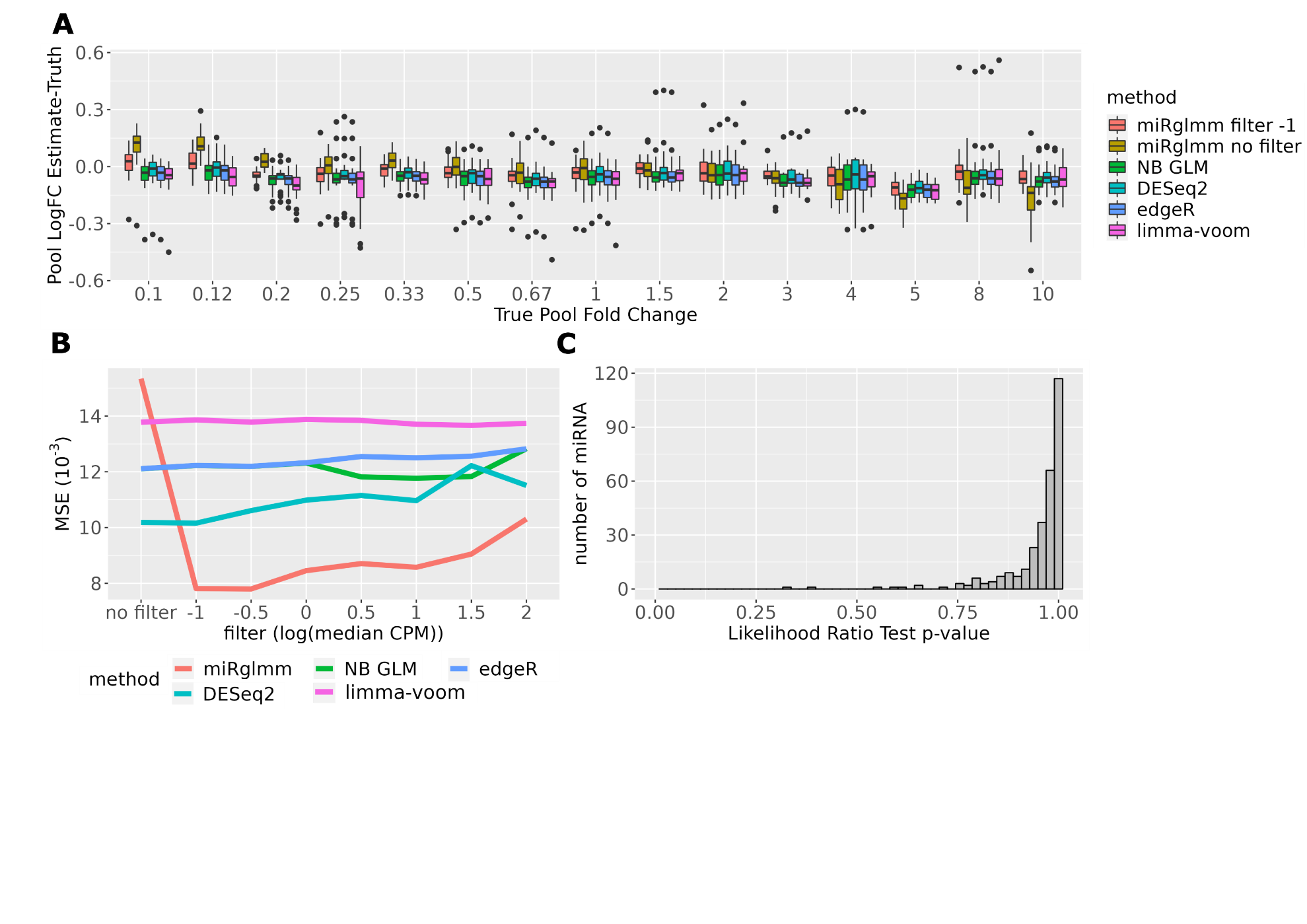


Fig. 5: Panel A displays the differences in log Fold Change (logFC) estimates comparing Pool B expression to Pool A and known true values by True Pool Fold Change and method. Panel B displays the effect of filtering on the performance of each method: miRglmm after filtering out isomiRs with log(median CPM) < -1, miRglmm with no filtering and 4 aggregation methods (with no filtering). Filtering results in a dramatic decrease in Mean Squared Error (MSE) when using miRglmm, but there is little change in performance after filtering for the aggregation methods. Panel C shows that there are no miRNA with differential isomiR usage (as measured via a Likelihood Ratio Test p-value) in the synthetic ERCC data.


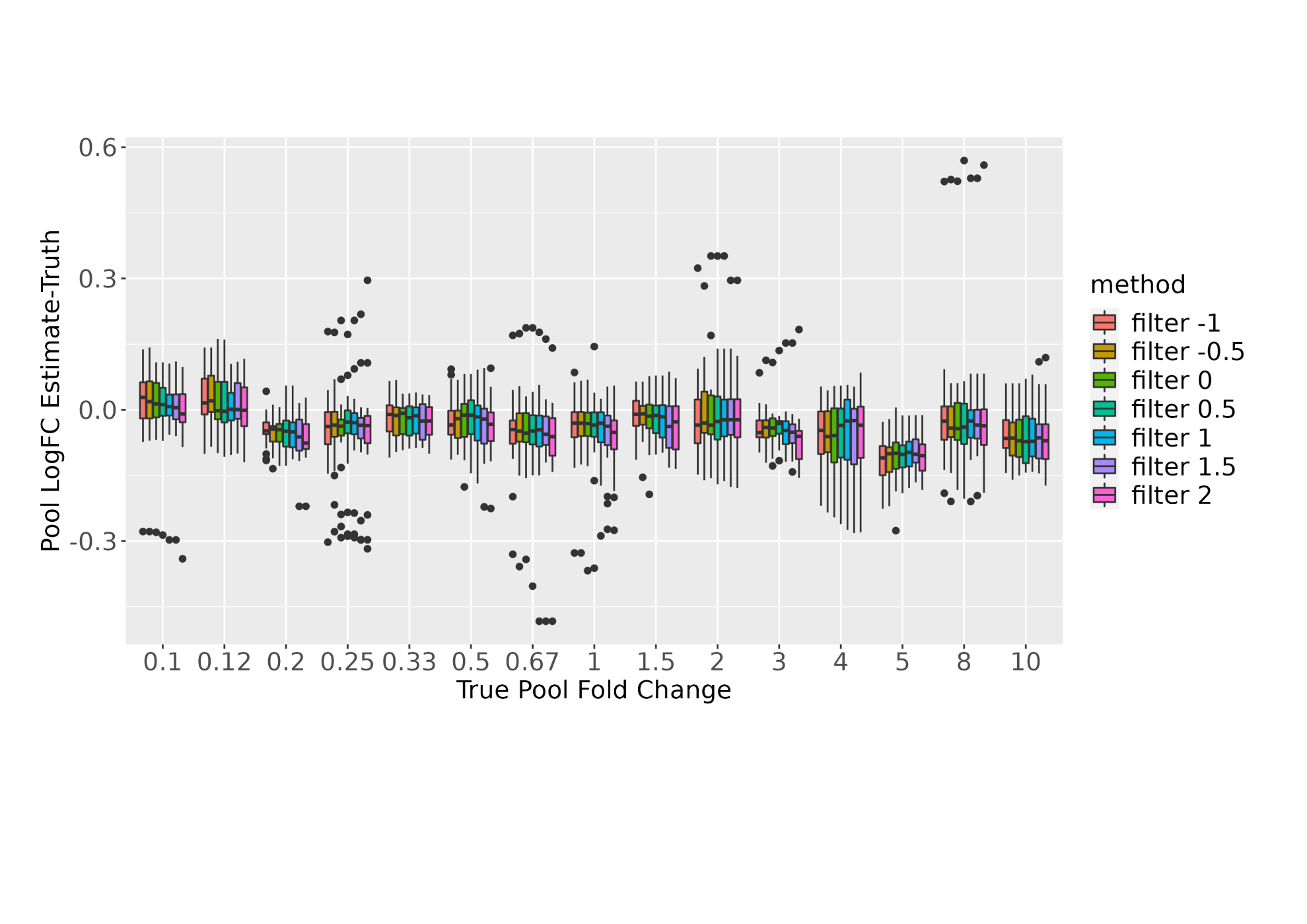


Fig. S6: The performance of miRglmm after a variety of isomiR filters based on log(median CPM) expression appears fairly uniform. Log fold change (logFC) estimates of Pool B expression compared to Pool A expression are compared to known true values for each miRNA and summarized within True Pool Fold Change group.

Table S30: Comparing performance by miRglmm filters and comparing to aggregation methods when random slope effect removed

| method | MSE (10^-3^) | number of  miRNA estimated | minimum number  of sequences | maximum number  of sequences | coverage  proportion |
| --- | --- | --- | --- | --- | --- |
| miRglmm filter -1 | 7.80 | 303 | 7 | 40 | 0.98 |
| miRglmm filter -0.5 | 7.79 | 303 | 6 | 33 | 0.98 |
| miRglmm filter 0 | 8.44 | 303 | 4 | 30 | 0.97 |
| miRglmm filter 0.5 | 8.67 | 302 | 2 | 24 | 0.98 |
| miRglmm filter 1 | 8.56 | 301 | 2 | 22 | 0.98 |
| miRglmm filter 1.5 | 9.05 | 301 | 2 | 18 | 0.99 |
| miRglmm filter 2 | 10.29 | 300 | 2 | 15 | 0.99 |
| miRglmm no filter | 14.80 | 303 | 12 | 263 | 0.97 |
| miRglmnb | 12.11 | 303 | NA | NA | 0.97 |
| DESeq2 | 10.19 | 303 | NA | NA | 0.99 |
| edgeR | 12.11 | 303 | NA | NA | NA |
| limma-voom | 13.78 | 303 | NA | NA | 0.99 |


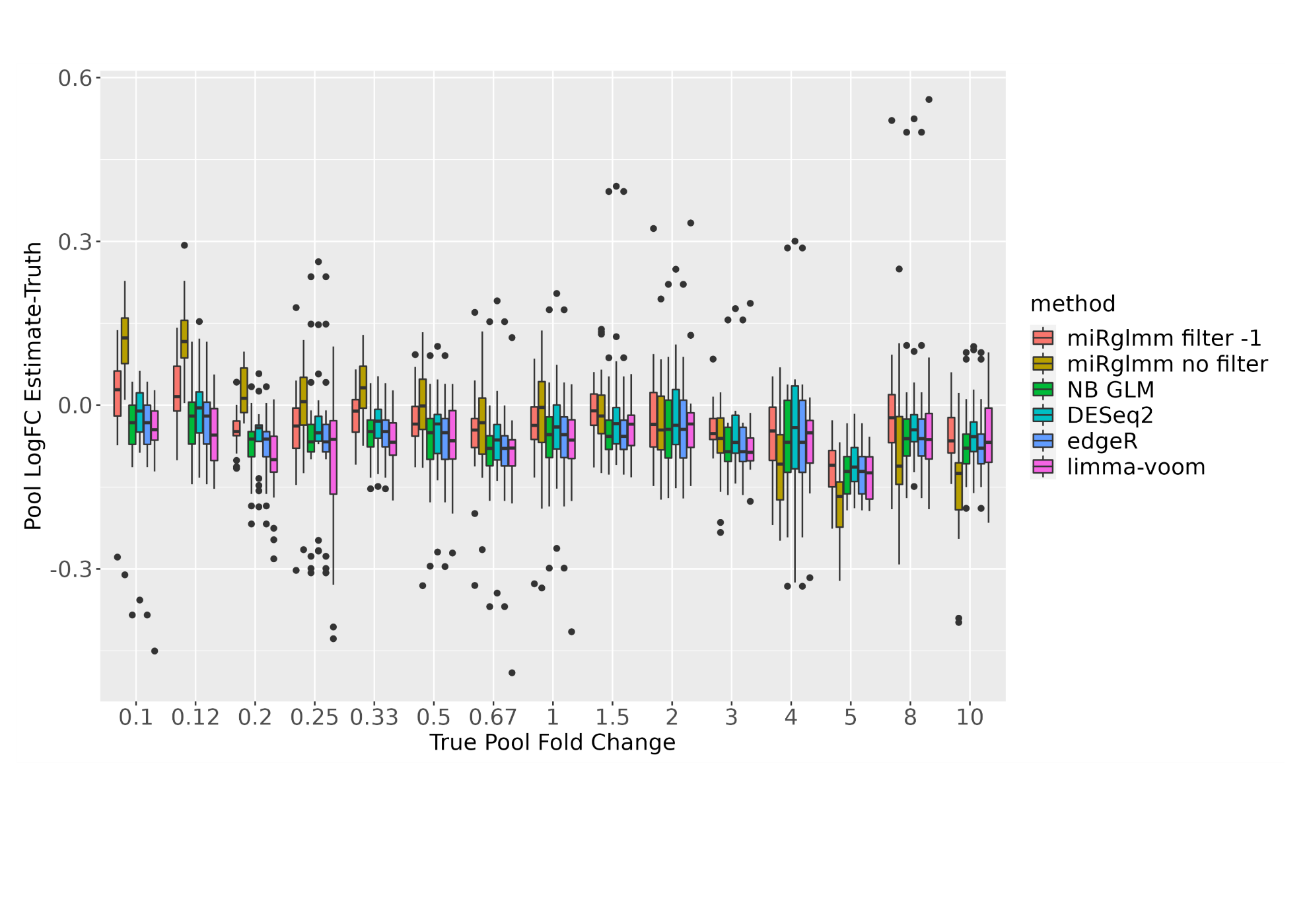


Fig. S7: The performance of miRglmm without the random Pool effect for isomiR is compared to the performance of aggregation methods. Log fold change (logFC) estimates of Pool B expression compared to Pool A expression are compared to known true values for each miRNA and summarized within each True Pool Fold Change group.

Table S31: Comparing performance of miRglmm to aggregation methods without adjustment for Lab

|  | MSE (10^-3^) | coverage proportion | null variance (10^-3^) | FC=2 variance* (10^-3^) | TPR | TNR | AUC |
| --- | --- | --- | --- | --- | --- | --- | --- |
| miRglmm | 8.02 | 1.00 | 4.67 | 6.65 | 0.58 | 1.00 | 1.00 |
| miRglmm-Poisson | 32.13 | 0.99 | 12.95 | 38.16 | 0.76 | 1.00 | 0.98 |
| NB GLM | 12.40 | 0.99 | 4.94 | 8.36 | 0.84 | 0.97 | 0.97 |
| DESeq2 | 12.80 | 1.00 | 11.00 | 8.95 | 0.81 | 1.00 | 1.00 |
| edgeR | 12.27 | NA | 4.83 | 8.35 | 0.75 | 1.00 | 1.00 |
| limma-voom | 16.96 | 1.00 | 9.75 | 13.97 | 0.69 | 1.00 | 0.99 |

MSE: Mean Squared Error, DE: differentially expressed, TPR: True Positive Rate, TNR: True Negative Rate, AUC: Area under ROC Curve, NA: not applicable, *chosen as representative of all values of truth


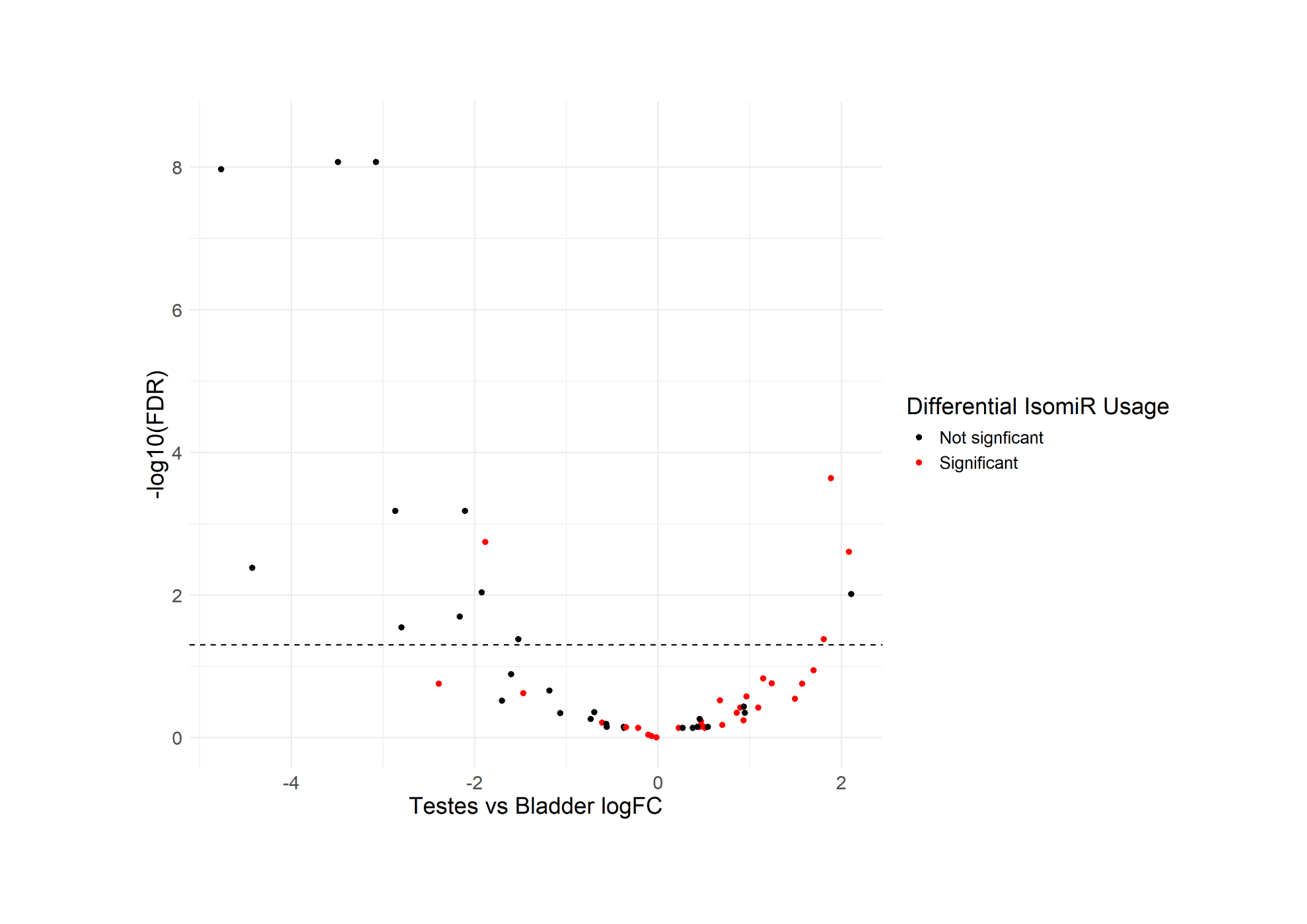


Fig. S8: Volcano plot of differentially expressed miRNA between testes and bladder as identified by miRglmm. Differential isomiR usage detected by miRglmm is indicated by color.

Table S32: Log Fold Change estimates (with 95% CI) of differentially expressed miRNA as identified by miRglmm with other DE method estimates for comparison.

| miRNA | miRglmm | NB GLM | DESeq2 | edgeR logFC | limma-voom |
| --- | --- | --- | --- | --- | --- |
| hsa-miR-127-3p* | 2.08 (0.95, 3.22) | 1.5 (0.61, 2.39) | 1.53 (0.71, 2.36) | 1.51 (NA, NA) | 1.94 (0.73, 3.15) |
| hsa-miR-140-3p* | 1.88 (1.03, 2.74) | 1.96 (1.24, 2.68) | 1.94 (1.25, 2.62) | 1.96 (NA, NA) | 2.08 (0.96, 3.2) |
| hsa-miR-192-5p | 2.11 (0.78, 3.43) | 2.53 (1.48, 3.59) | 2.18 (1.35, 3) | 2.53 (NA, NA) | 2.1 (0.76, 3.45) |
| hsa-miR-25-3p* | 1.81 (0.43, 3.18) | 2.02 (0.93, 3.12) | 1.79 (0.95, 2.63) | 2.02 (NA, NA) | 1.95 (0.55, 3.34) |
| hsa-miR-1-3p | -2.16 (-3.64, -0.69) | -1.83 (-2.99, -0.67) | -2.89 (-4.07, -1.71) | -1.83 (NA, NA) | -2.35 (-3.78, -0.91) |
| hsa-miR-126-3p | -2.8 (-4.81, -0.79) | -0.32 (-1.95, 1.31) | -1.51 (-2.88, -0.14) | -0.35 (NA, NA) | -2.63 (-4.18, -1.08) |
| hsa-miR-143-3p | -4.77 (-6.27, -3.26) | -2.46 (-3.91, -1.01) | -4.06 (-5.58, -2.54) | -2.46 (NA, NA) | -4.82 (-6.44, -3.2) |
| hsa-miR-200c-3p | -4.43 (-6.97, -1.89) | -2.37 (-4.43, -0.31) | -2.61 (-4.43, -0.79) | -2.4 (NA, NA) | -3.77 (-5.57, -1.97) |
| hsa-miR-24-3p | -1.53 (-2.7, -0.36) | -0.48 (-1.53, 0.57) | -1.26 (-2.23, -0.3) | -0.46 (NA, NA) | -1.18 (-2.44, 0.08) |
| hsa-miR-27b-3p* | -1.89 (-2.89, -0.89) | -0.95 (-1.82, -0.07) | -1.73 (-2.66, -0.8) | -0.88 (NA, NA) | -1.65 (-2.79, -0.51) |
| hsa-miR-28-3p | -2.11 (-3.14, -1.07) | -1.73 (-2.23, -1.22) | -1.88 (-2.63, -1.14) | -1.6 (NA, NA) | -1.51 (-2.51, -0.5) |
| hsa-miR-30a-3p | -1.93 (-3.12, -0.73) | -2.06 (-2.98, -1.15) | -2.47 (-3.51, -1.43) | -1.86 (NA, NA) | -1.81 (-3, -0.62) |
| hsa-miR-378a-3p | -2.87 (-4.27, -1.47) | -1.33 (-2.73, 0.06) | -2.25 (-3.41, -1.09) | -1.34 (NA, NA) | -2.99 (-4.44, -1.55) |
| hsa-miR-378c | -3.08 (-3.08, -3.08) | -1.5 (-2.84, -0.15) | -2.26 (-3.37, -1.15) | -1.5 (NA, NA) | -2.9 (-4.26, -1.54) |
| hsa-miR-378d | -3.49 (-3.5, -3.49) | -1.96 (-3.38, -0.54) | -2.64 (-3.78, -1.5) | -1.99 (NA, NA) | -3.07 (-4.47, -1.66) |

*indicates miRNA with differential isomiR usage

Table S33: Log Fold Change estimates (with 95% CI) for 10 miRNA with largest differential isomiR usage

| miRNA | miRglmm | NB GLM | DESeq2 | edgeR logFC | limma-voom |
| --- | --- | --- | --- | --- | --- |
| hsa-let-7a-5p | 0.48 (-0.94, 1.91) | 1.07 (0.31, 1.84)* | 1.33 (0.51, 2.15)* | 1.07 (NA, NA) | 1.04 (-0.09, 2.17) |
| hsa-let-7b-5p | 0.93 (-1.05, 2.91) | 2.24 (1.01, 3.48)* | 3.44 (2.16, 4.72)* | 2.24 (NA, NA) | 1.88 (0.36, 3.39)* |
| hsa-let-7c-5p | 1.24 (-0.03, 2.51) | 1.32 (0.52, 2.13)* | 2.1 (1.1, 3.1)* | 1.32 (NA, NA) | 1.3 (0.16, 2.43)* |
| hsa-let-7g-5p | 0.46 (-0.82, 1.74) | 1.29 (0.46, 2.12)* | 0.99 (0.13, 1.86)* | 1.29 (NA, NA) | 0.81 (-0.37, 2) |
| hsa-miR-146b-5p | 0.51 (-1.64, 2.67) | 2 (0.64, 3.35)* | 2.2 (0.99, 3.4)* | 2 (NA, NA) | 2.19 (0.38, 4)* |
| hsa-miR-181a-5p | 0.7 (-1.15, 2.55) | -1.07 (-2.26, 0.12) | -0.66 (-1.64, 0.33) | -1.07 (NA, NA) | 0.16 (-1.41, 1.73) |
| hsa-miR-181b-5p | 1.49 (-0.39, 3.37) | -0.03 (-1.3, 1.24) | 0.51 (-0.56, 1.59) | -0.03 (NA, NA) | 1.17 (-0.53, 2.88) |
| hsa-miR-191-5p | 1.7 (0.16, 3.23) | 0.61 (-0.72, 1.95) | 1.27 (0.18, 2.35)* | 0.61 (NA, NA) | 1.63 (0.17, 3.08)* |
| hsa-miR-29a-3p | -0.02 (-1.81, 1.77) | 0.05 (-1.56, 1.65) | 0.76 (-0.53, 2.05) | 0.05 (NA, NA) | 1.39 (-0.22, 3.01) |
| hsa-miR-30e-5p | -1.47 (-3.18, 0.24) | 0.29 (-1.03, 1.6) | -1.18 (-1.99, -0.36)* | 0.29 (NA, NA) | -0.85 (-2.38, 0.69) |

*indicates significant differential expression


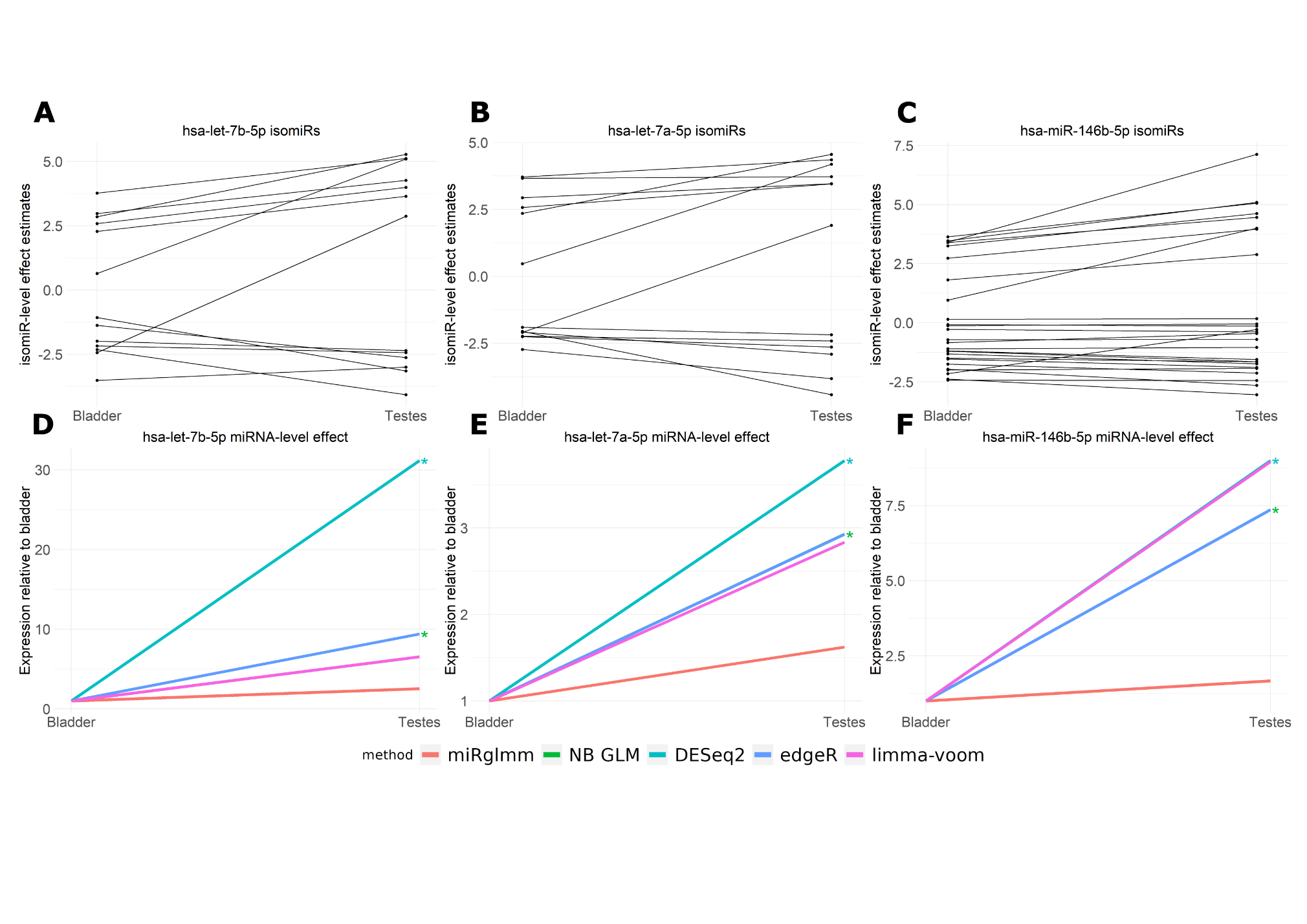


Fig. S9: IsomiR variability in miRNA with differential isomiR usage (panels A-C) and associated differences in miRNA-level differential expression estimates between methods (panels D-F). Stars indicate significant miRNA-level differential expression. NB GLM and edgeR provide nearly identical estimates for all miRNA.


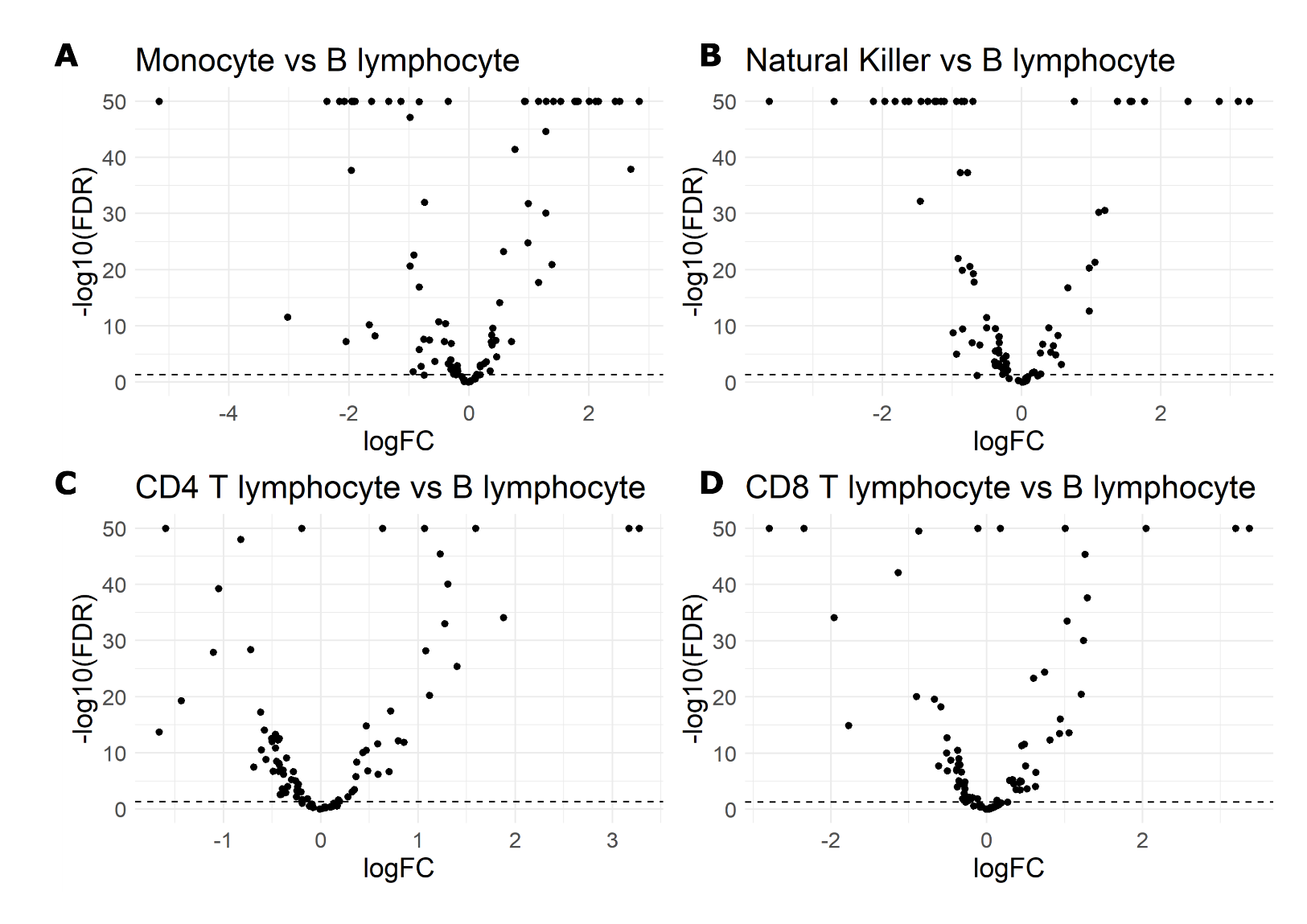


Fig. S10: Volcano plot of differentially expressed miRNA between monocytes (panel A), natural killer cells (panel B), CD4+ T lymphocytes (panel C), CD8+ T lymphocytes (panel D) and B lymphocytes as identified by miRglmm. All miRNA have differential isomiR usage across 5 cell types. Y-axis capped at 50 to allow visualization of non-significant range.


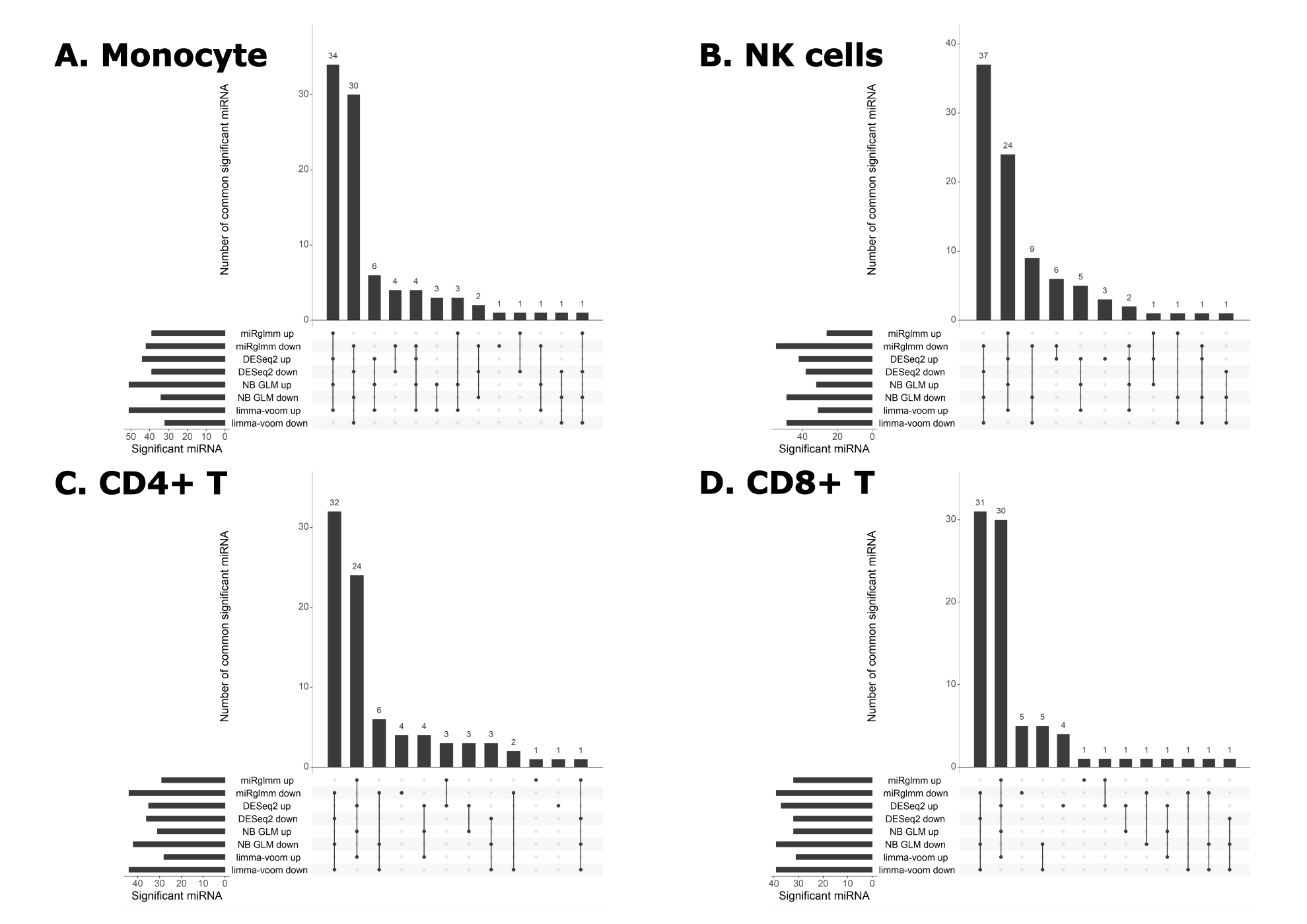


Fig. S11: Upset plots show agreement between miRNA called differentially expressed between methods. edgeR not included due to not providing contrast-specific inference.

Table S34: Log Fold Change (logFC) estimates (with 95% CI, where applicable) for all methods in miRNA found to be differentially expressed by miRglmm only

| miRNA | miRglmm | miRglmnb | DESeq2 | edgeR | limmavoom | contrast | logFC via qPCR (32) |
| --- | --- | --- | --- | --- | --- | --- | --- |
| miR-19b-3p | -0.3 (-0.45, -0.15) | 0.12 (-0.06, 0.3) | -0.03 (-0.19, 0.14) | 0.12 | 0.1 (-0.08, 0.28) | Monocyte vs B lymphocyte | -0.49 |
| miR-181a-3p | -0.49 (-0.67, -0.31) | -0.15 (-0.32, 0.02) | -0.1 (-0.27, 0.08) | -0.15 | -0.12 (-0.3, 0.05) | CD4 T lymphocyte vs B lymphocyte | -0.28 |
| miR-191-5p | -0.42 (-0.54, -0.31) | -0.02 (-0.18, 0.14) | 0.01 (-0.15, 0.17) | -0.02 | -0.12 (-0.27, 0.03) | CD4 T lymphocyte vs B lymphocyte | -1.32 |
| miR-32-5p | 0.28 (0.09, 0.48) | -0.06 (-0.23, 0.11) | 0.03 (-0.14, 0.19) | -0.06 | -0.09 (-0.28, 0.1) | CD4 T lymphocyte vs B lymphocyte | -0.62 |
| miR-423-5p | -0.42 (-0.68, -0.15) | -0.23 (-0.51, 0.05) | -0.13 (-0.42, 0.16) | -0.23 | -0.24 (-0.53, 0.05) | CD4 T lymphocyte vs B lymphocyte | -0.28 |
| miR-98-5p | -0.34 (-0.5, -0.17) | 0.04 (-0.15, 0.24) | 0.1 (-0.09, 0.28) | 0.04 | -0.02 (-0.22, 0.18) | CD4 T lymphocyte vs B lymphocyte | -0.21 |
| hmiR-191-5p | -0.34 (-0.46, -0.23) | -0.06 (-0.22, 0.11) | 0.01 (-0.15, 0.18) | -0.06 | -0.06 (-0.21, 0.1) | CD8 T lymphocyte vs B lymphocyte | -1.25 |
| miR-26a-5p | -0.12 (-0.21, -0.03) | -0.01 (-0.1, 0.07) | 0.08 (-0.04, 0.2) | -0.01 | -0.02 (-0.11, 0.07) | CD8 T lymphocyte vs B lymphocyte | -0.07 |
| miR-30b-5p | 0.34 (0.19, 0.5) | -0.08 (-0.24, 0.09) | 0.03 (-0.14, 0.2) | -0.08 | -0.07 (-0.24, 0.09) | CD8 T lymphocyte vs B lymphocyte | 0.21 |
| miR-340-5p | -0.27 (-0.53, -0.01) | -0.23 (-0.51, 0.06) | -0.16 (-0.41, 0.09) | -0.23 | -0.25 (-0.51, 0.01) | CD8 T lymphocyte vs B lymphocyte | -1.59 |
| miR-423-3p | -0.11 (-0.11, -0.11) | -0.05 (-0.18, 0.09) | 0.04 (-0.11, 0.19) | -0.05 | -0.05 (-0.19, 0.08) | CD8 T lymphocyte vs B lymphocyte | -0.28 |
| miR-98-5p | -0.29 (-0.46, -0.12) | 0.14 (-0.06, 0.34) | 0.18 (0, 0.37) | 0.14 | 0.06 (-0.15, 0.26) | CD8 T lymphocyte vs B lymphocyte | -0.35 |


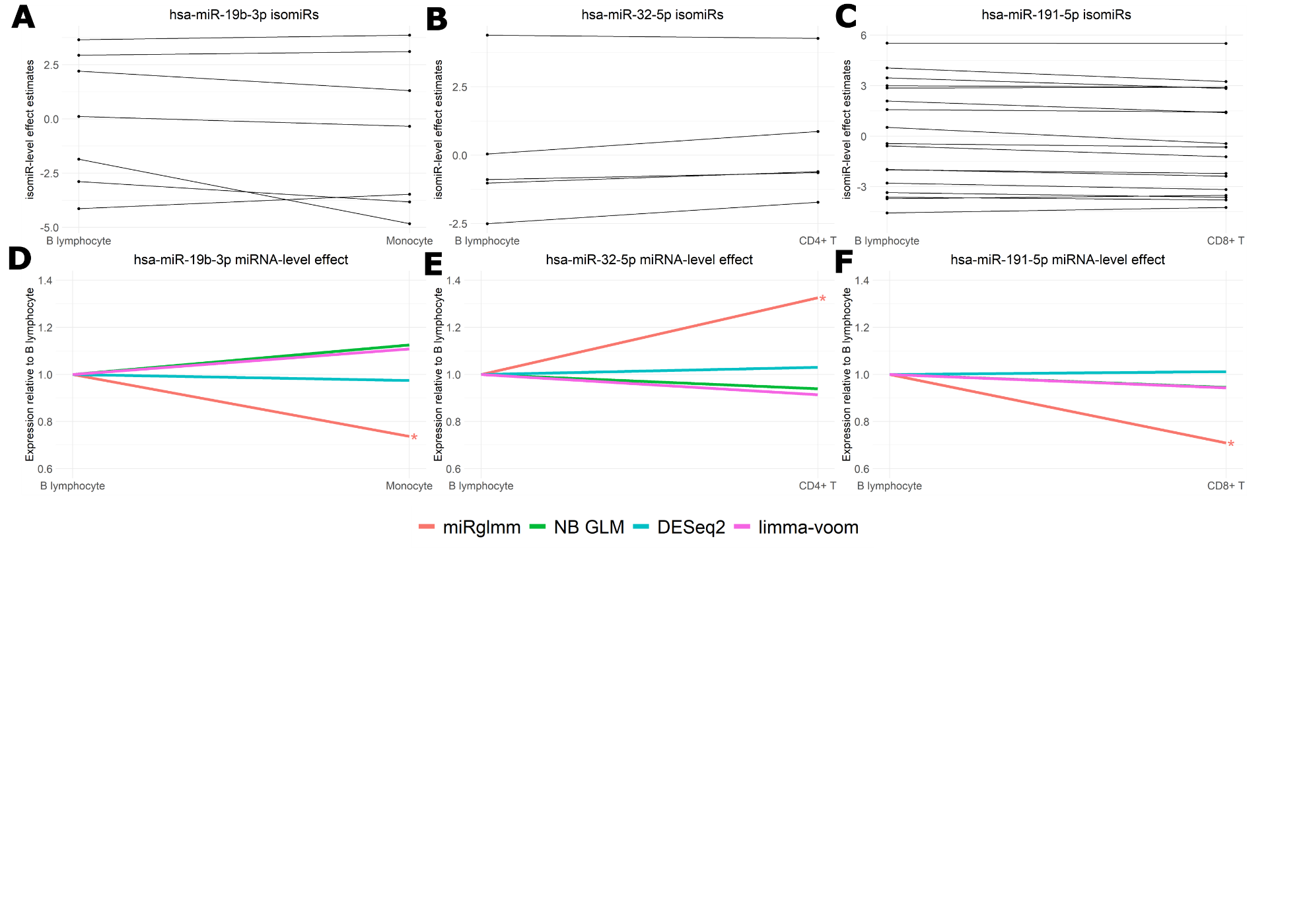


Fig. S12: IsomiR variability in 3 of the miRNA identified by miRglmm only as being differentially expressed (panels A-C) and associated miRNA-level differential expression estimates for all methods (panels D-F). Stars indicate significant miRNA-level differential expression. edgeR not included since it does not provide contrast-specific inference.


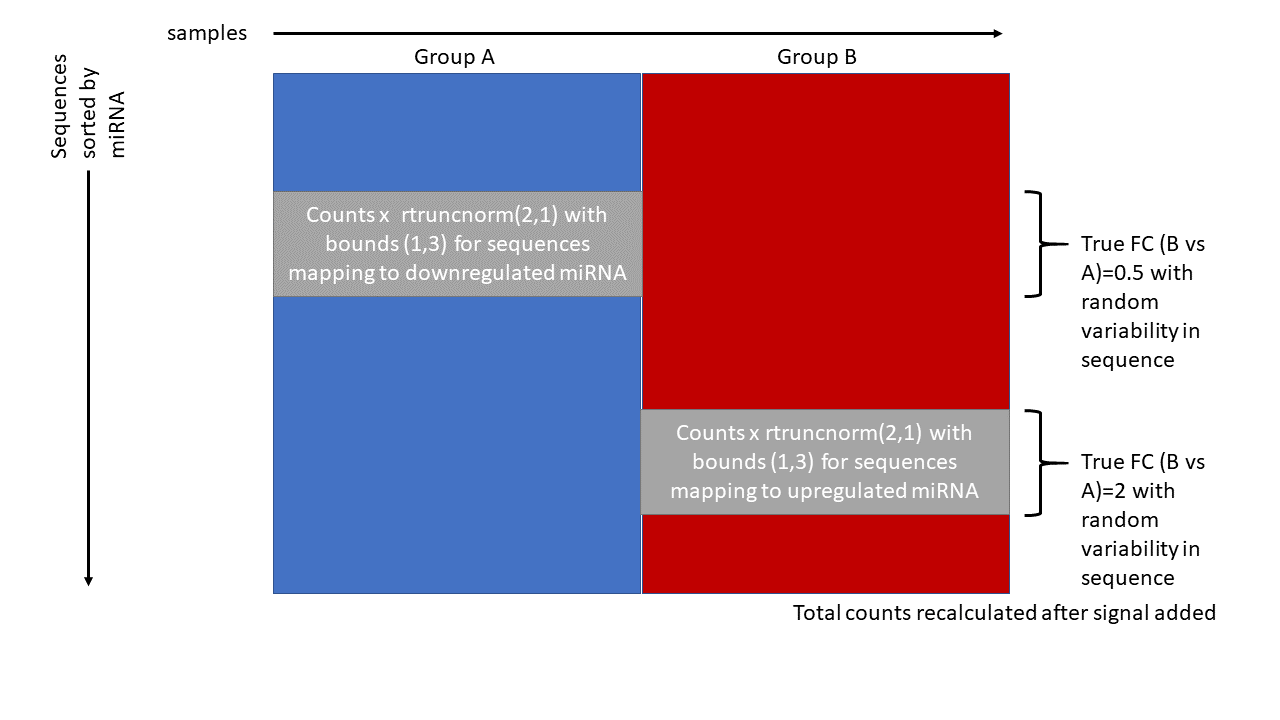


Fig. S13: Inducing an artificial group effect with differential isomiR usage into monocyte miRNA-seq data. The entire procedure from random splitting of samples into groups is repeated 100 times.


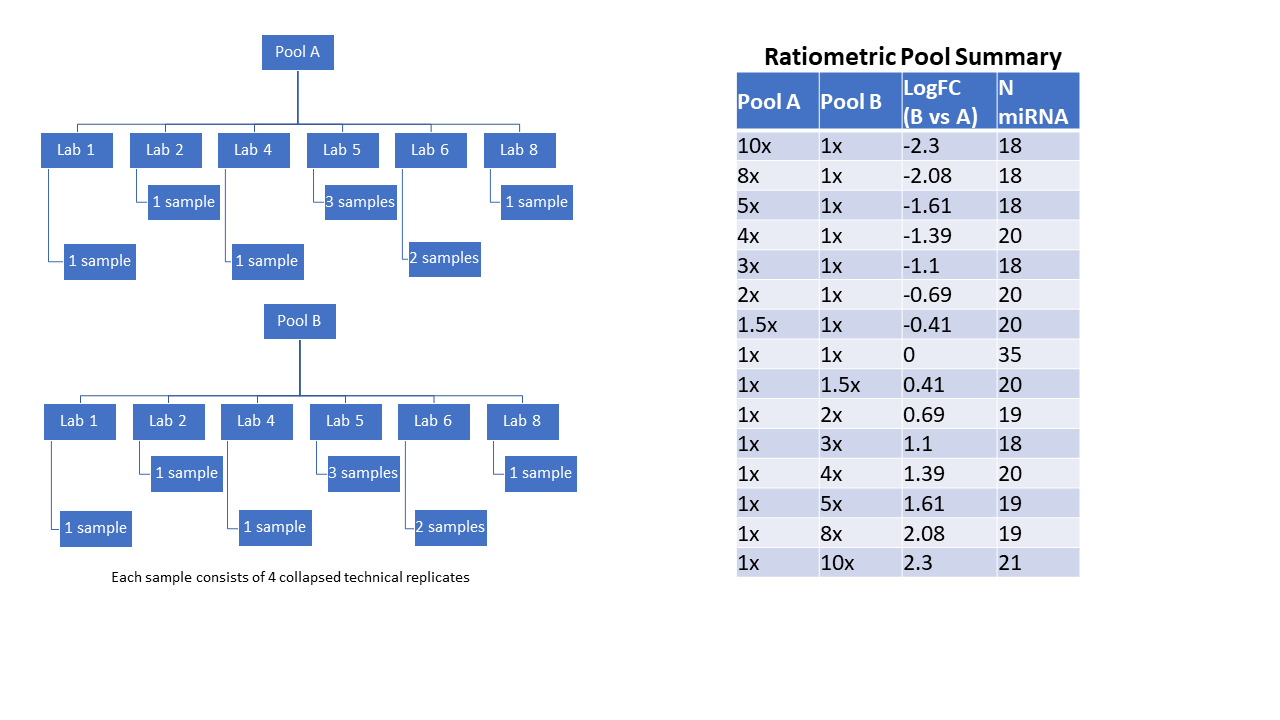


Fig. S14: ERCC experimental design. Samples from two pools were generated by six laboratories. Each synthetic miRNA was added to Pool A and Pool B at one of fifteen possible ratios.


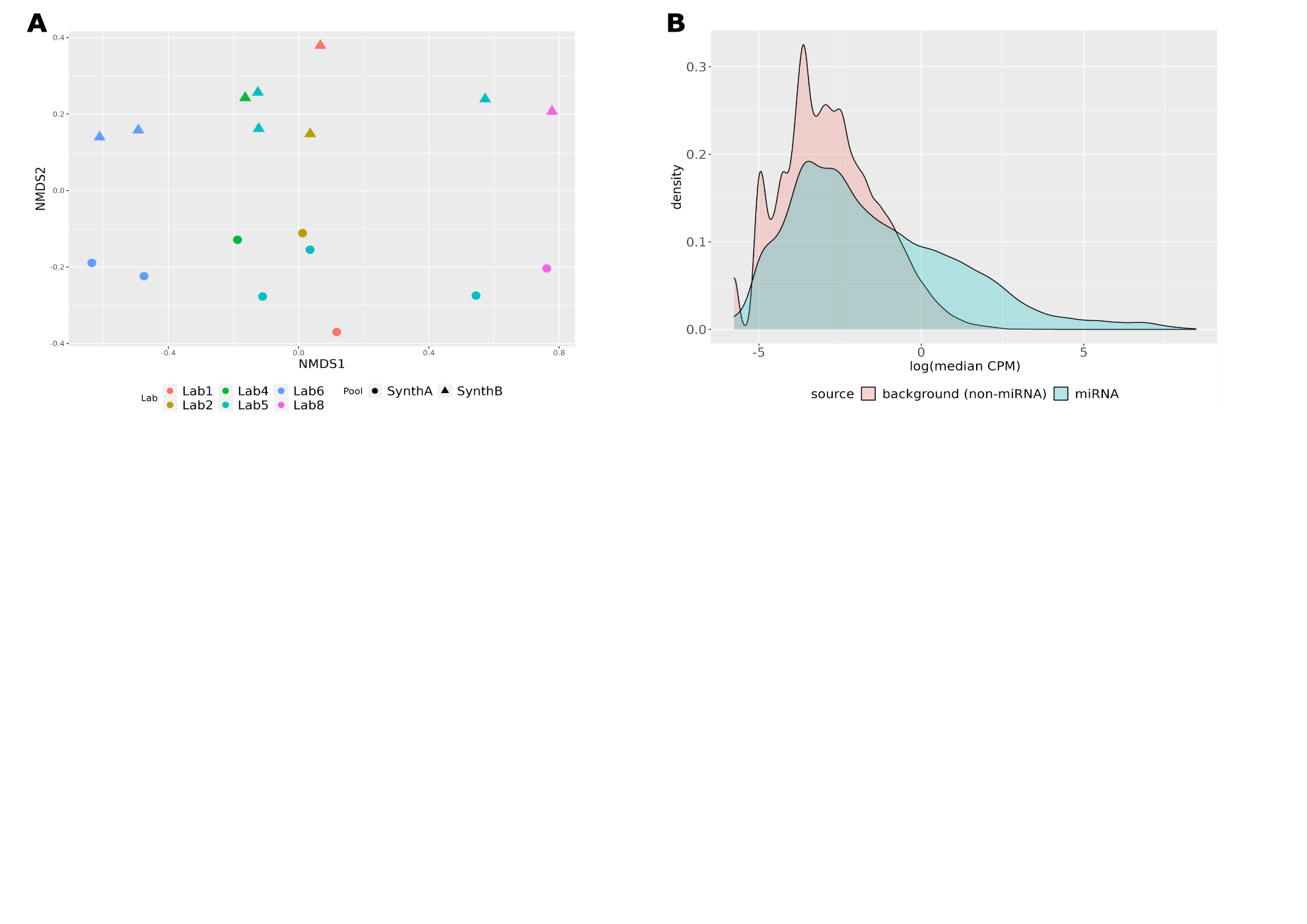


Fig. S15: Non-metric multidimensional rescaling (NMDS) of raw counts reveals separation of samples based on laboratory along the first dimension and separation based on Pool along the second dimension (panel A). Density plots comparing the distributions of expression between sequences mapped to miRNA and sequences not mapped to miRNA can be used to define a threshold for retaining sequences with expression above background levels (panel B).
