# Supplementary material for: miRglmm: a generalized linear mixed model of isomiR-level counts improves estimation of miRNA-level differential expression and uncovers variable differential expression between isomiRs
Source: Genome Biol. 2025 Apr 22;26:102. doi: 10.1186/s13059-025-03549-y (PMC12016310; doi:10.1186/s13059-025-03549-y)
Supplement: Supplementary file 2 — Additional File 2: miRglmm software package vignette [file 13059_2025_3549_MOESM2_ESM.docx]

# A generalized linear mixed model for miRNA-seq data

#### [Andrea M. Baran and Matthew N. McCall](http://mnmccall.com)

#### 2025-01-07

# Introduction

In this vignette we demonstrate how to analyze microRNA-seq data at the isomiR-level using the miRglmm package. This package begins with a SummarizedExperiment object containing aligned isomiR-level read counts. The alignment can be performed by either miRge or srabench. We provide instructions for both alignment algorithms, starting with the fastq files and concluding with the SummarizedExperiment object to be used as input to the miRglmm function for modeling. Each step in the analysis is briefly described and carried out by one or more functions implemented in this package. Each of these functions has its own help file describing it in detail; here we focus on the analysis as a whole.

# Experimental Design

Our example data set consists of 6 monocyte samples. Fastq files for these samples can be accessed through the sequence read archive (SRA), via this link:

# miRge processing and alignment

miRge 3.0 can be downloaded ([here](https://github.com/mhalushka/miRge3.0/)) and descriptions of miRge3.0 parameters are provided. To process the 6 monocytes samples from [SRP110505](https://www.ncbi.nlm.nih.gov/sra?term=SRP110505) included as example data in this package: #### To execute miRge3.0, change the directory to SRR_folder (Alternatively, one could provide absolute paths) miRge3.0 -s SRR5755870.fastq.gz,SRR5755996.fastq.gz,SRR5755961.fastq.gz,SRR5755982.fastq.gz,SRR5755821.fastq.gz,SRR5756084.fastq.gz -gff -bam -trf -lib miRge3_Lib -on human -db mirbase -o OutputDir -mEC -ks 20 -ke 20

## Load miRNA counts for exact.miRNA (canonical sequences) and isomiR.miRNAs (isomiR sequences) and create SummarizedExperiment

The annotated smallRNA results can be located in folder miRge.YEAR-MM-DD_HR_MM_SS (default output folder with date and time). To load canonical and isomiR sequences can be loaded from mapped.csv file inside the ouput folder as shown below:

# Creating Summarized Experiment from miRge3.0 output

library(dplyr)

mapped <- read.csv("mapped.csv")

mapped_miRNAs <- select(mapped,-c("annotFlag","hairpin.miRNA","mature.tRNA","primary.tRNA","snoRNA","rRNA","ncrna.others","mRNA"))

mapped_miRNAs <- mapped_miRNAs[c((mapped_miRNAs$exact.miRNA != '') | (mapped_miRNAs$isomiR.miRNA != '')),]

mapped_miRNAs$match<- with(mapped_miRNAs, ifelse(exact.miRNA != '', "exact miRNA", "isomiR miRNA"))

mapped_miRNAs$miRNA<- with(mapped_miRNAs, ifelse(exact.miRNA != '', exact.miRNA, isomiR.miRNA))

rownames(mapped_miRNAs) <- NULL

# Preparing Assay

expressionCounts <- mapped_miRNAs[,6:ncol(mapped_miRNAs)-2]

# Preparing rowData

miRNA_data <- mapped_miRNAs[c('Sequence','match','miRNA')]

names(miRNA_data)[names(miRNA_data) == 'Sequence'] <- 'uniqueSequence'

# Preparing colData

meta <- mapped <- read.csv("annotation.report.csv", header=TRUE)

meta <- meta[c(1,6,7)]

colnames(meta) <- c('sample_id', 'filtered_miRNA_reads', 'unique_miRNAs')

# The sample groups for differential expression should accordingly be mentioned by the researcher as a new column.

rownames(meta) <- meta$sample_id

# Preparing Summarized Experiment Object

mirge_SE <- SummarizedExperiment(assays=list(counts=expressionCounts), colData=meta, rowData = miRNA_data)

# sRNAbench processing and alignment

After installing sRNAbench ([instructions here](https://github.com/bioinfoUGR/sRNAtoolbox)), you can directly download and align SRA files from your terminal. To process the 6 monocytes samples from [SRP110505](https://www.ncbi.nlm.nih.gov/sra?term=SRP110505) included as example data in this package:

sRNAbench input=SRR5755870 output=srnabench/SRR5755870 adapter=TGGAATTCTCGGGTGCCAAGGG microRNA=hsa isoMiR=true

sRNAbench input=SRR5755996 output=srnabench/SRR5755996 adapter=TGGAATTCTCGGGTGCCAAGGG microRNA=hsa isoMiR=true

sRNAbench input=SRR5755961 output=srnabench/SRR5755961 adapter=TGGAATTCTCGGGTGCCAAGGG microRNA=hsa isoMiR=true

sRNAbench input=SRR5755982 output=srnabench/SRR5755982 adapter=TGGAATTCTCGGGTGCCAAGGG microRNA=hsa isoMiR=true

sRNAbench input=SRR5755821 output=srnabench/SRR5755821 adapter=TGGAATTCTCGGGTGCCAAGGG microRNA=hsa isoMiR=true

sRNAbench input=SRR5756084 output=srnabench/SRR5756084 adapter=TGGAATTCTCGGGTGCCAAGGG microRNA=hsa isoMiR=true

We then run sRNAde to generate a matrix of counts:

sRNAde input=srnabench/ output=sRNAde_result grpString=SRR5755870:SRR5755996:SRR5755961:SRR5755982:SRR5755821:SRR5756084 readIso=true colReadIso=5 minExprReadIso=1

The resulting isomiR count matrix file, microRNAannotation_minExpr1_RC.mat, can be found in the sRNAde_result directory. To load it into a SummarizedExperiment object:

library("SummarizedExperiment")

input_df <- read.delim("sRNAde_result/microRNAannotation_minExpr1_RC.mat", check.names = FALSE)

input_data <- as.matrix(input_df[,-1])

input_row_data <- strsplit(input_df$name, "\\|")

formated_row_data = data.frame(uniqueSequence = sapply(input_row_data, function(x) x[1]),

miRNA = sapply(input_row_data, function(x) x[2]))

input_col_data <- strsplit(colnames(input_df), "\\|")

formated_col_data = data.frame(sampleID = sapply(input_col_data[-1], function(x) x[1]),

group = sapply(input_col_data[-1], function(x) x[2]))

SummarizedExperiment(assays=list(counts=input_data),

rowData=formated_row_data,

colData=formated_col_data,

checkDimnames=TRUE)

# Statistical modeling with miRglmm

library("miRglmm")

library("SummarizedExperiment")

data("se_example")

The example data and corresponding annotation are stored in a SummarizedExperiment object:

show(se_example)

## class: SummarizedExperiment

## dim: 101 6

## metadata(0):

## assays(1): counts

## rownames: NULL

## rowData names(3): uniqueSequence match miRNA

## colnames(6): SRR5755870 SRR5755996 ... SRR5755821 SRR5756084

## colData names(14): sample_id organ ... sequencer flagged

str(assay(se_example))

## Formal class 'dgCMatrix' [package "Matrix"] with 6 slots

## ..@ i : int [1:271] 1 2 6 9 10 16 18 19 26 28 ...

## ..@ p : int [1:7] 0 33 88 132 188 230 271

## ..@ Dim : int [1:2] 101 6

## ..@ Dimnames:List of 2

## .. ..$ : NULL

## .. ..$ : chr [1:6] "SRR5755870" "SRR5755996" "SRR5755961" "SRR5755982" ...

## ..@ x : num [1:271] 299 7 34 288 4 3 2 14 58 1 ...

## ..@ factors : list()

The column names of the assay in the SummarizedExperiment object must correspond to unique sample IDs:

head(colnames(assay(se_example)))

## [1] "SRR5755870" "SRR5755996" "SRR5755961" "SRR5755982" "SRR5755821" "SRR5756084"

The colData contain information about each sample:

colData(se_example)

## DataFrame with 6 rows and 14 columns

## sample_id organ total_input_reads trimmed_reads filtered_miRNA_reads unique_miRNAs

## <character> <character> <numeric> <numeric> <numeric> <numeric>

## SRR5755870 SRR5755870 blood cell populatio.. 3475202 3223065 810250 451

## SRR5755996 SRR5755996 blood cell populatio.. 11701090 10574645 5389504 731

## SRR5755961 SRR5755961 blood cell populatio.. 6031197 5548812 2288477 566

## SRR5755982 SRR5755982 blood cell populatio.. 8082720 6960649 3164519 631

## SRR5755821 SRR5755821 blood cell populatio.. 1910166 1608911 665464 416

## SRR5756084 SRR5756084 blood cell populatio.. 2739415 2415155 1569563 500

## percent_miRNAs cell_tissue study_id biocluster sample_category library_prep sequencer flagged

## <numeric> <character> <numeric> <numeric> <character> <character> <character> <logical>

## SRR5755870 0.233152 Monocyte 89 0 cell_type Illumina TruSeq smal.. HiSeq 2500 FALSE

## SRR5755996 0.460598 Monocyte 89 0 cell_type Illumina TruSeq smal.. HiSeq 2500 FALSE

## SRR5755961 0.379440 Monocyte 89 0 cell_type Illumina TruSeq smal.. HiSeq 2500 FALSE

## SRR5755982 0.391517 Monocyte 89 0 cell_type Illumina TruSeq smal.. HiSeq 2500 FALSE

## SRR5755821 0.348380 Monocyte 89 0 cell_type Illumina TruSeq smal.. HiSeq 2500 FALSE

## SRR5756084 0.572956 Monocyte 89 0 cell_type Illumina TruSeq smal.. HiSeq 2500 FALSE

The rowData contain information about each isomiR, including its sequence (“uniqueSequence”) and the miRNA (“miRNA”) it aligned to:

rowData(se_example)

## DataFrame with 101 rows and 3 columns

## uniqueSequence match miRNA

## <character> <factor> <character>

## 1 CAAATCCATGCAAAACTGA exact miRNA hsa-miR-19b-3p

## 2 GTACAGTACTGTGATAACTGA exact miRNA hsa-miR-101-3p

## 3 TGAGGTAGTAGATTGTA exact miRNA hsa-let-7f-5p

## 4 TGAGGTAGTAGATTGTATAG.. exact miRNA hsa-let-7f-5p

## 5 TGTGCAAATCCATGCA exact miRNA hsa-miR-19b-3p

## ... ... ... ...

## 97 TACTGTGATAACTGAA exact miRNA hsa-miR-101-3p

## 98 CAGTACTGTGATAACTGAAGG exact miRNA hsa-miR-101-3p

## 99 CAGTACTGTGATAACTGAAGGA exact miRNA hsa-miR-101-3p

## 100 ACTGTGATAACTGAAG exact miRNA hsa-miR-101-3p

## 101 GCTGTGCAAATCCATGCAAA.. exact miRNA hsa-miR-19b-3p

## Fitting the GLMM model

A SummarizedExperiment object (for example, se_example) is input into miRglmm, along with a vector specifying primary fixed effect of interest for Differential Expression Analysis (DEA), col_group. This can be 2 or more level categorical variable. For miRglmm to appropriately model the random effects for sample and sequence, the columns names of the assay object in the input SummarizedExperiment object (for example, assay(se_example)) must be the unique sample IDs. There must also be elements of rowData (for example, rowData(se_example)) that are called “uniqueSequence” and “miRNA”, which indicate the unique sequence/isomiR identifier, and the unique miRNA identifiers, respectively.

Additional input arguments include: min_med_lcpm (default -1), ncores (default 1), and adjust_var (default NA). min_med_lcpm specifies a threshold on median(log(CPM)) expression for retaining sequences to include in the analysis. Larger values retain fewer (more highly expressed) sequences/isomiRs in the analysis. ncores is used to specify the number of cores available to run the algorithm, and the analysis of individual miRNA will be run in parallel across cores if ncores>1. adjust_var can be used to specify an adjustment variable (continuous or categorical) that will be used as an additional fixed effect in the miRglmm model.

fit = miRglmm(se = se_example, col_group = rep(c("A","B"), c(3,3)))

## [1] "running non-parallel"

## hsa-miR-19b-3p

## hsa-miR-101-3p

## hsa-let-7f-5p

## Output of the GLMM model

The output of miRglmm is a list of glmerMod model fit objects called “miRglmm” with length equal to the number of miRNA modelled. The elements of the list are named by miRNA (based on unique entries in rowData(se)$miRNA). This allows flexible extraction of any model fit parameter of interest for downstream analysis.

An additional output of miRglmm is another list of glmerMod model fits called “miRglmm_reduced”. This is equivalent in size and structure to the “miRglmm” list output, but it is model fit objects after removal of the col_group|sequence random effect, and can be used to perform a likelihood ratio test for the significance of the random slope parameter.

## Downstream analyses

The fixed effects can be obtained using the following commands:

all_coeff=sapply(fit[["miRglmm"]], "fixef")

The effect of primary interest (col_group) can be obtained by looking at that specific row in the fit object:

library(stringr)

idx=which(str_detect(rownames(all_coeff), "col_group"))

coeff_full=data.frame("full"=all_coeff[idx,])

rownames(coeff_full)=colnames(all_coeff)

Plots of the primary fixed effect of interest, with overlaid isomiR-level random effects, can be plotted for a specified miRNA. Here we use hsa-miR-100-5p as an example:

miRNA_plot=names(fit[["miRglmm"]])[1]

f1=fit[["miRglmm"]][[miRNA_plot]]

#pull out individual sequence estimates

idx=which(str_detect(colnames(ranef(f1)$sequence), "col_group"))

idx_fixed=which(str_detect(names(fixef(f1)), "col_group"))

groupA=fixef(f1)[1]+ranef(f1)$sequence$`(Intercept)`-fixef(f1)[1]

groupB=fixef(f1)[1]+ranef(f1)$sequence$`(Intercept)`+ranef(f1)$sequence[,idx]+fixef(f1)[idx_fixed]-fixef(f1)[1]

groupA=data.frame("estimate"=groupA, "sequence"=rownames(ranef(f1)$sequence))

groupB=data.frame("estimate"=groupB, "sequence"=rownames(ranef(f1)$sequence))

groupA$Pool="Bladder"

groupB$Pool="Testes"

comb_df=rbind(groupA, groupB)

#pull out fixed effect estimates

groupA=fixef(f1)[1]-fixef(f1)[1]

groupB=fixef(f1)[1]+fixef(f1)[idx_fixed]-fixef(f1)[1]

groupA=data.frame("estimate"=groupA)

groupB=data.frame("estimate"=groupB)

groupA$Pool="Bladder"

groupB$Pool="Testes"

comb_df_overall=rbind(groupA, groupB)

ggplot(comb_df, aes(x=Pool, y=estimate, group=sequence, color="miRglmm isomiR estimates (random effects)"))+geom_line(alpha=0.5)+geom_point(alpha=0.5)+

geom_line(data=comb_df_overall, aes(x=Pool, y=estimate, group=1, color="miRglmm estimates (fixed effects)"), size=2)+

xlab("tissue")+

scale_y_continuous(breaks=c(-4, -2, 0, 2), labels=round(exp(c(-4,-2,0,2)),3))+

ylab(paste('Expression relative to', '\n', 'average bladder expression'))+scale_x_discrete(expand=c(0.05,0))+xlab('')+

scale_color_manual(name="", values=c("miRglmm estimates (fixed effects)"="red", "miRglmm isomiR estimates (random effects)"="black"))+

theme(legend.position="bottom", legend.direction="horizontal")


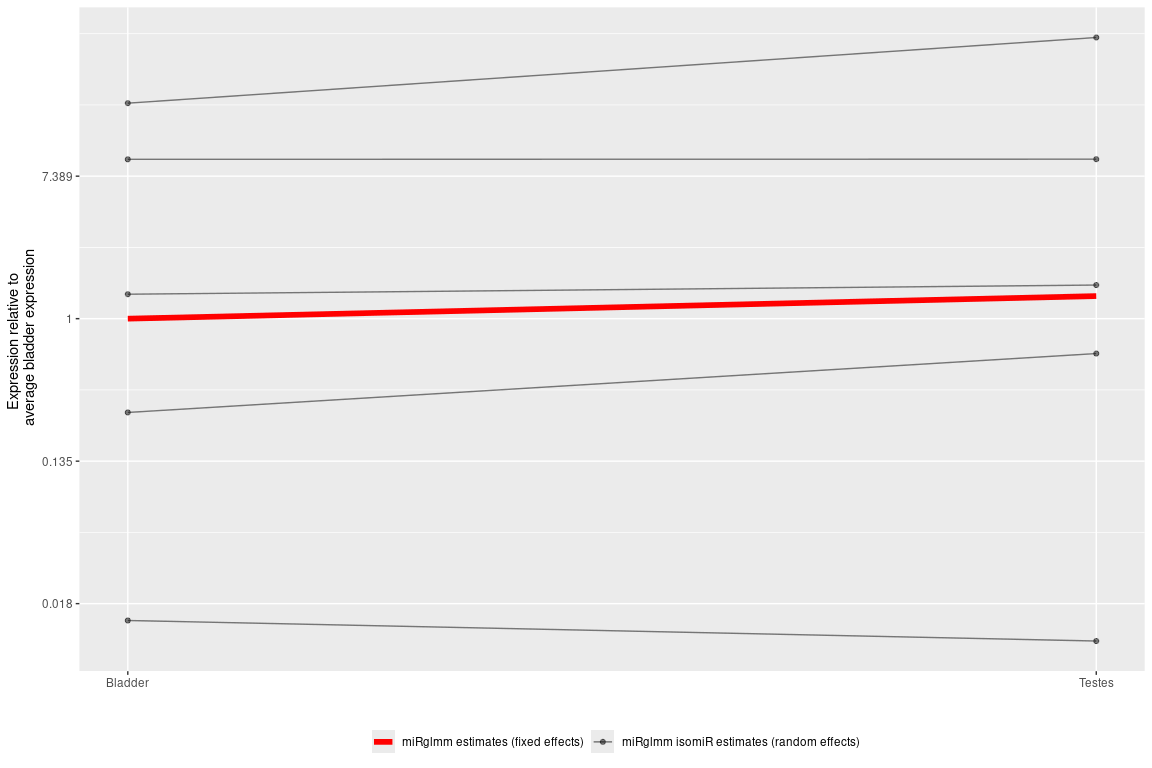


The Standard Error (SE), and 95% Wald-based Confidence Intervals, for the primary fixed effect of interest can be found via:

all_SE=sapply(fit[["miRglmm"]], "vcov")

idx1=which(str_detect(rownames(all_SE[[1]]), "col_group")==TRUE)

idx2=which(str_detect(colnames(all_SE[[1]]), "col_group")==TRUE)

SE_vec=data.frame('SE_full'=sapply(all_SE, function(x) sqrt(x[idx1,idx2])))

rownames(SE_vec)=names(all_SE)

z_alpha=qnorm(1-(1-0.95)/2)

LL_mat=coeff_full-z_alpha*SE_vec

UL_mat=coeff_full+z_alpha*SE_vec

Wald p-values can be found with the following:

all_pvals=sapply(fit[["miRglmm"]], function(f) summary(f)$coefficients[, "Pr(>|z|)"])

idx=which(str_detect(rownames(all_pvals), "col_group"))

pval_full=data.frame("full"=all_pvals[idx,])

rownames(pval_full)=colnames(all_pvals)

A likelihood-ratio test for the col_group|sequence effect can be performed via:

uniq_miRNA=intersect(names(fit[["miRglmm"]]), names(fit[["miRglmm_reduced"]]))

LRTp=data.frame("LRTp"=sapply(uniq_miRNA, function(row) anova(fit[["miRglmm"]][[row]], fit[["miRglmm_reduced"]][[row]])$`Pr(>Chisq)`[2]))

rownames(LRTp)=uniq_miRNA

The distribution of the LRT p-values can be plotted via:

library(ggplot2)

ggplot(LRTp, aes(x=LRTp))+geom_histogram(color="black", fill="gray", bins=50)+xlab('Likelihood Ratio Test p-value')+ylab('number of miRNA')


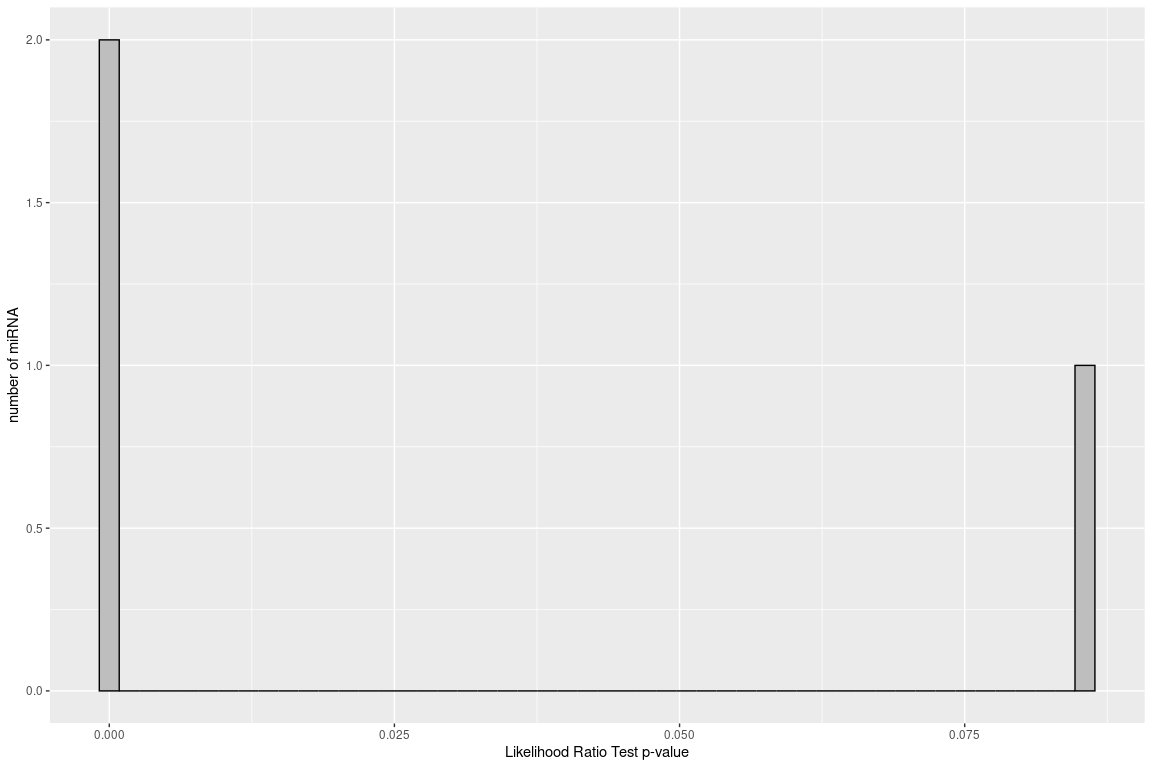
The following code can be used to obtain the variance components from the (full) miRglmm model:

uniq_miRNA=names(fit[["miRglmm"]])

var_comp=data.frame('random_int_sample_var'=sapply(uniq_miRNA, function(row) VarCorr(fit[["miRglmm"]][[row]])$sample_labels[1,1]))

var_comp2=data.frame('random_int_seq_var'=sapply(uniq_miRNA, function(row) VarCorr(fit[["miRglmm"]][[row]])$sequence[1,1]))

var_comp=transform(merge(var_comp, var_comp2, by='row.names', all=T), row.names=Row.names, Row.names=NULL)

var_comp2=data.frame('random_slope_seq_var'=sapply(uniq_miRNA, function(row) VarCorr(fit[["miRglmm"]][[row]])$sequence[2,2]))

var_comp=transform(merge(var_comp, var_comp2, by='row.names', all=T), row.names=Row.names, Row.names=NULL)

# Session Info

sessionInfo()

## R version 4.1.1 (2021-08-10)

## Platform: x86_64-pc-linux-gnu (64-bit)

## Running under: Red Hat Enterprise Linux Server 7.9 (Maipo)

##

## Matrix products: default

## BLAS: /gpfs/fs1/sfw2/r/4.1.1/b1/lib64/R/lib/libRblas.so

## LAPACK: /gpfs/fs1/sfw2/r/4.1.1/b1/lib64/R/lib/libRlapack.so

##

## locale:

## [1] LC_CTYPE=en_US.UTF-8 LC_NUMERIC=C LC_TIME=en_US.UTF-8 LC_COLLATE=en_US.UTF-8

## [5] LC_MONETARY=en_US.UTF-8 LC_MESSAGES=en_US.UTF-8 LC_PAPER=en_US.UTF-8 LC_NAME=C

## [9] LC_ADDRESS=C LC_TELEPHONE=C LC_MEASUREMENT=en_US.UTF-8 LC_IDENTIFICATION=C

##

## attached base packages:

## [1] parallel stats4 stats graphics grDevices utils datasets methods base

##

## other attached packages:

## [1] knitr_1.37 miRglmm_0.99.0 doParallel_1.0.17 iterators_1.0.14

## [5] foreach_1.5.2 SummarizedExperiment_1.24.0 Biobase_2.54.0 GenomicRanges_1.46.1

## [9] GenomeInfoDb_1.30.1 IRanges_2.28.0 S4Vectors_0.32.3 BiocGenerics_0.40.0

## [13] MatrixGenerics_1.6.0 matrixStats_0.61.0 lme4_1.1-35.5 Matrix_1.6-4

## [17] reshape2_1.4.4 forcats_0.5.1 stringr_1.5.0 dplyr_1.1.4

## [21] purrr_1.0.2 readr_2.1.2 tidyr_1.3.1 tibble_3.2.1

## [25] ggplot2_3.5.1 tidyverse_1.3.1 devtools_2.4.5 usethis_2.1.6

## [29] rmarkdown_2.11

##

## loaded via a namespace (and not attached):

## [1] minqa_1.2.4 colorspace_2.0-2 ellipsis_0.3.2 rprojroot_2.0.2 XVector_0.34.0

## [6] fs_1.5.2 rstudioapi_0.13 farver_2.1.0 remotes_2.4.2 fansi_1.0.2

## [11] lubridate_1.8.0 xml2_1.3.3 codetools_0.2-18 splines_4.1.1 cachem_1.0.6

## [16] pkgload_1.3.2 jsonlite_1.7.3 nloptr_1.2.2.2 broom_0.7.12 dbplyr_2.1.1

## [21] shiny_1.7.1 compiler_4.1.1 httr_1.4.2 backports_1.4.1 assertthat_0.2.1

## [26] fastmap_1.1.0 cli_3.6.1 later_1.3.0 htmltools_0.5.2 prettyunits_1.1.1

## [31] tools_4.1.1 gtable_0.3.0 glue_1.6.2 GenomeInfoDbData_1.2.7 Rcpp_1.0.13

## [36] jquerylib_0.1.4 cellranger_1.1.0 vctrs_0.6.5 nlme_3.1-155 xfun_0.29

## [41] ps_1.6.0 rvest_1.0.2 mime_0.12 miniUI_0.1.1.1 lifecycle_1.0.3

## [46] zlibbioc_1.40.0 MASS_7.3-55 scales_1.3.0 hms_1.1.1 promises_1.2.0.1

## [51] yaml_2.3.4 memoise_2.0.1 sass_0.4.0 stringi_1.7.12 highr_0.9

## [56] desc_1.4.2 boot_1.3-28 pkgbuild_1.3.1 rlang_1.1.2 pkgconfig_2.0.3

## [61] bitops_1.0-7 evaluate_0.15 lattice_0.20-45 labeling_0.4.2 htmlwidgets_1.5.4

## [66] processx_3.8.0 tidyselect_1.2.0 plyr_1.8.6 magrittr_2.0.3 R6_2.5.1

## [71] generics_0.1.3 profvis_0.3.7 DelayedArray_0.20.0 DBI_1.1.2 pillar_1.9.0

## [76] haven_2.4.3 withr_2.5.0 RCurl_1.98-1.6 modelr_0.1.8 crayon_1.5.1

## [81] utf8_1.2.2 tzdb_0.4.0 urlchecker_1.0.1 grid_4.1.1 readxl_1.3.1

## [86] callr_3.7.3 reprex_2.0.1 digest_0.6.29 xtable_1.8-4 httpuv_1.6.5

## [91] munsell_0.5.0 bslib_0.3.1 sessioninfo_1.2.2
